# Ecological and evolutionary drivers of haemoplasma infection and bacterial genotype sharing in a Neotropical bat community

**DOI:** 10.1111/mec.15422

**Published:** 2020-04-21

**Authors:** Daniel J. Becker, Kelly A. Speer, Alexis M. Brown, M. Brock Fenton, Alex D. Washburne, Sonia Altizer, Daniel G. Streicker, Raina K. Plowright, Vladimir E. Chizhikov, Nancy B. Simmons, Dmitriy V. Volokhov

**Affiliations:** ^1^ Department of Biology Indiana University Bloomington IN USA; ^2^ Center for the Ecology of Infectious Disease University of Georgia Athens GA USA; ^3^ Richard Gilder Graduate School American Museum of Natural History New York NY USA; ^4^ Department of Invertebrate Zoology National Museum of Natural History Smithsonian Institution Washington DC USA; ^5^ Center for Conservation Genomics Smithsonian Conservation Biology Institute National Zoological Park Washington DC USA; ^6^ Department of Ecology and Evolution Stony Brook University Stony Brook NY USA; ^7^ Department of Biology Western University London ON Canada; ^8^ Department of Microbiology and Immunology Montana State University Bozeman MT USA; ^9^ Odum School of Ecology University of Georgia Athens GA USA; ^10^ MRC–University of Glasgow Centre for Virus Research Glasgow UK; ^11^ Institute of Biodiversity, Animal Health and Comparative Medicine University of Glasgow Glasgow UK; ^12^ Center for Biologics Evaluation and Research, Food and Drug Administration Silver Spring MD USA; ^13^ Department of Mammalogy Division of Vertebrate Zoology American Museum of Natural History New York NY USA

**Keywords:** 16S rRNA, bacterial zoonosis, cophylogeny, host shifts, host specificity, *Mycoplasma*, parasite sharing

## Abstract

Most emerging pathogens can infect multiple species, underlining the importance of understanding the ecological and evolutionary factors that allow some hosts to harbour greater infection prevalence and share pathogens with other species. However, our understanding of pathogen jumps is based primarily around viruses, despite bacteria accounting for the greatest proportion of zoonoses. Because bacterial pathogens in bats (order Chiroptera) can have conservation and human health consequences, studies that examine the ecological and evolutionary drivers of bacterial prevalence and barriers to pathogen sharing are crucially needed. Here were studied haemotropic *Mycoplasma* spp. (i.e., haemoplasmas) across a species‐rich bat community in Belize over two years. Across 469 bats spanning 33 species, half of individuals and two‐thirds of species were haemoplasma positive. Infection prevalence was higher for males and for species with larger body mass and colony sizes. Haemoplasmas displayed high genetic diversity (21 novel genotypes) and strong host specificity. Evolutionary patterns supported codivergence of bats and bacterial genotypes alongside phylogenetically constrained host shifts. Bat species centrality to the network of shared haemoplasma genotypes was phylogenetically clustered and unrelated to prevalence, further suggesting rare—but detectable—bacterial sharing between species. Our study highlights the importance of using fine phylogenetic scales when assessing host specificity and suggests phylogenetic similarity may play a key role in host shifts not only for viruses but also for bacteria. Such work more broadly contributes to increasing efforts to understand cross‐species transmission and the epidemiological consequences of bacterial pathogens.

## 1 INTRODUCTION

Most pathogens that cause disease in humans, domestic animals and wildlife are capable of infecting multiple host species (Woolhouse, Taylor, & Haydon, 2001). However, predicting which hosts maintain pathogens and identifying their role in cross‐species transmission can be challenging, as many hosts can be infected but not play key roles in the reservoir community (Fenton, Streicker, Petchey, & Pedersen, 2015). Pathogen jumps between species depend on infection prevalence in the donor host, transmission opportunities between donor and recipient species, and suitability of the recipient host for pathogen replication (Plowright et al., 2017). Each of these steps can be shaped by ecological and evolutionary factors (VanderWaal & Ezenwa, 2016). For example, small‐bodied species can have greater competence, the ability to transmit new infections, than larger species (Downs, Schoenle, Han, Harrison, & Martin, 2019), and host switching is often constrained by phylogeny, owing to similarity in immunological barriers to pathogen replication between closely related species (Streicker et al., 2010). Identifying the ecological and evolutionary factors that allow some species to harbour greater prevalence and have facilitated pathogen sharing can improve our general understanding of disease emergence (Fountain‐Jones et al., 2018). Examining evolutionary associations between hosts and pathogens can further uncover factors favouring host shifts versus codivergence and assess the frequency of cross‐species transmission (Geoghegan, Duchêne, & Holmes, 2017).

Given the public health and agricultural burdens of many zoonotic pathogens such as avian influenza viruses, henipaviruses and lyssaviruses, many investigations of pathogen prevalence and emergence focus on viruses (Geoghegan et al., 2017; Olival et al., 2017). However, more zoonoses are caused by bacteria than other pathogen taxa (Han, Kramer, & Drake, 2016), and bacterial pathogens can negatively impact newly infected host species (e.g., *Mycoplasma galliscepticum*, a poultry pathogen, caused rapid population declines in wild house finches; Hochachka & Dhondt, 2000). More attention to bacteria and their propensity for host specificity versus generalism is accordingly important for understanding whether factors that govern cross‐species transmission of viruses can be extended to other pathogens (Bonneaud, Weinert, & Kuijper, 2019). Bacterial pathogens have been especially understudied for bats (Mühldorfer, 2013), in contrast to intensive studies of zoonotic viruses across the Chiroptera (Luis et al., 2015). However, many bacterial pathogens are probably important to both bat conservation and human health due to pathogenic effects on bats themselves as well as their zoonotic potential (Becker et al., 2018; Evans, Bown, Timofte, Simpson, & Birtles, 2009).

To determine the ecological and evolutionary drivers of bacterial prevalence and barriers to pathogen sharing, we focused on haemotropic *Mycoplasma* spp. (i.e., hemoplasmas) in a species‐rich bat community in Belize (Fenton et al., 2001; Herrera, Duncan, Clare, Fenton, & Simmons, 2018). The Neotropics have remarkable bat diversity owing to adaptive radiation in the Phyllostomidae (Gunnell & Simmons, 2012), producing a range of feeding strategies (e.g., frugivory, carnivory, sanguivory), body sizes and roost preferences (Monteiro & Nogueira, 2011). Haemoplasmas are facultative intracellular erythrocytic bacteria transmitted through direct contact (Cohen et al., 2018; Museux et al., 2009) and also possibly via arthropod vectors (Willi, Boretti, Meli, et al., 2007). Haemoplasmas can cause acute and chronic anaemia, especially for immunocompromised hosts; however, many animals develop inapparent infections and are asymptomatic (Messick, 2004). As *Mycoplasma* spp. lack many of the metabolic pathways associated with energy production and synthesis of cell components found in other bacteria, they are fully dependent on host cells (Citti & Blanchard, 2013). Haemoplasmas have therefore been described as mostly host specialists (Pitcher & Nicholas, 2005), yet interspecies and potentially zoonotic transmission can occur (Maggi et al., 2013; Willi, Boretti, Tasker, et al., 2007). Haemoplasmas are common and genetically diverse in bats (Di Cataldo, Kamani, Cevidanes, Msheliza, & Millán, 2020; Ikeda et al., 2017; Mascarelli et al., 2014; Volokhov, Becker, et al., 2017), which offers an ideal model system for identifying the ecological and evolutionary factors structuring bacterial infection risks within and between host species.

Many cross‐species comparisons of pathogen infection risks and sharing have used less‐diverse host communities (Johnson et al., 2012; VanderWaal, Atwill, Isbell, & McCowan, 2014) or global data sets of host–pathogen associations that can be limited by heterogeneous sampling effort and variation in pathogen detection methods (Dallas et al., 2019; Huang, Bininda‐Emonds, Stephens, Gittleman, & Altizer, 2014). Our focus on a widespread pathogen group in a highly diverse host community allowed us to capitalize on strong host trait variation while controlling for sampling effort and diagnostic methods (Becker, Crowley, Washburne, & Plowright, 2019; Han, Kramer, et al., 2016). Past work has also used host–pathogen networks to characterize contemporary or historical transmission at often coarse taxonomic scales (e.g., pathogen species complexes or genera; Blyton, Banks, Peakall, Lindenmayer, & Gordon, 2014; VanderWaal et al., 2014). However, as bat species can be infected by multiple haemoplasma genotypes, and because genotypes with ≥99% sequence identity of their 16S rRNA genes can represent different bacterial species (Volokhov, Hwang, Chizhikov, Danaceau, & Gottdenker, 2017; Volokhov, Simonyan, Davidson, & Chizhikov, 2012), focusing on genotypes provides finer‐scale resolution to determine the ecological and evolutionary features of species that facilitate pathogen sharing and to identify likely maintenance hosts of bacterial infections (Fountain‐Jones et al., 2018).

We investigated three questions about the relative contribution of ecological traits and evolutionary history to structuring infection patterns and pathogen sharing. First, what are the individual and ecological predictors of haemoplasma infection in a Neotropical bat community? Second, does the distribution of haemoplasma genotypes across the bat community map onto the bat phylogeny, as predicted by host–pathogen codivergence? Third, if genotype sharing among host species occurs, which host clades or traits best predict species ability to share pathogens? We predicted that ecological covariates such as ectoparasitism and large host colonies could increase bacteria risk through vector‐borne or density‐dependent transmission (McCallum, Barlow, & Hone, 2001; Willi, Boretti, Meli, et al., 2007). We also expected haemoplasma genotypes would be specific to particular host species but that more closely related bats would share haemoplasma genotypes, indicating phylogenetically restricted host shifts (Pitcher & Nicholas, 2005). However, ecological traits that increase the risk of pathogen exposure between species, such as occupying a greater diversity of roosting habitats, could also facilitate pathogen genotype sharing among less closely related hosts (McKee et al., 2019).

## 2 METHODS

### 2.1 Bat capture and sampling

**Figure 1:**
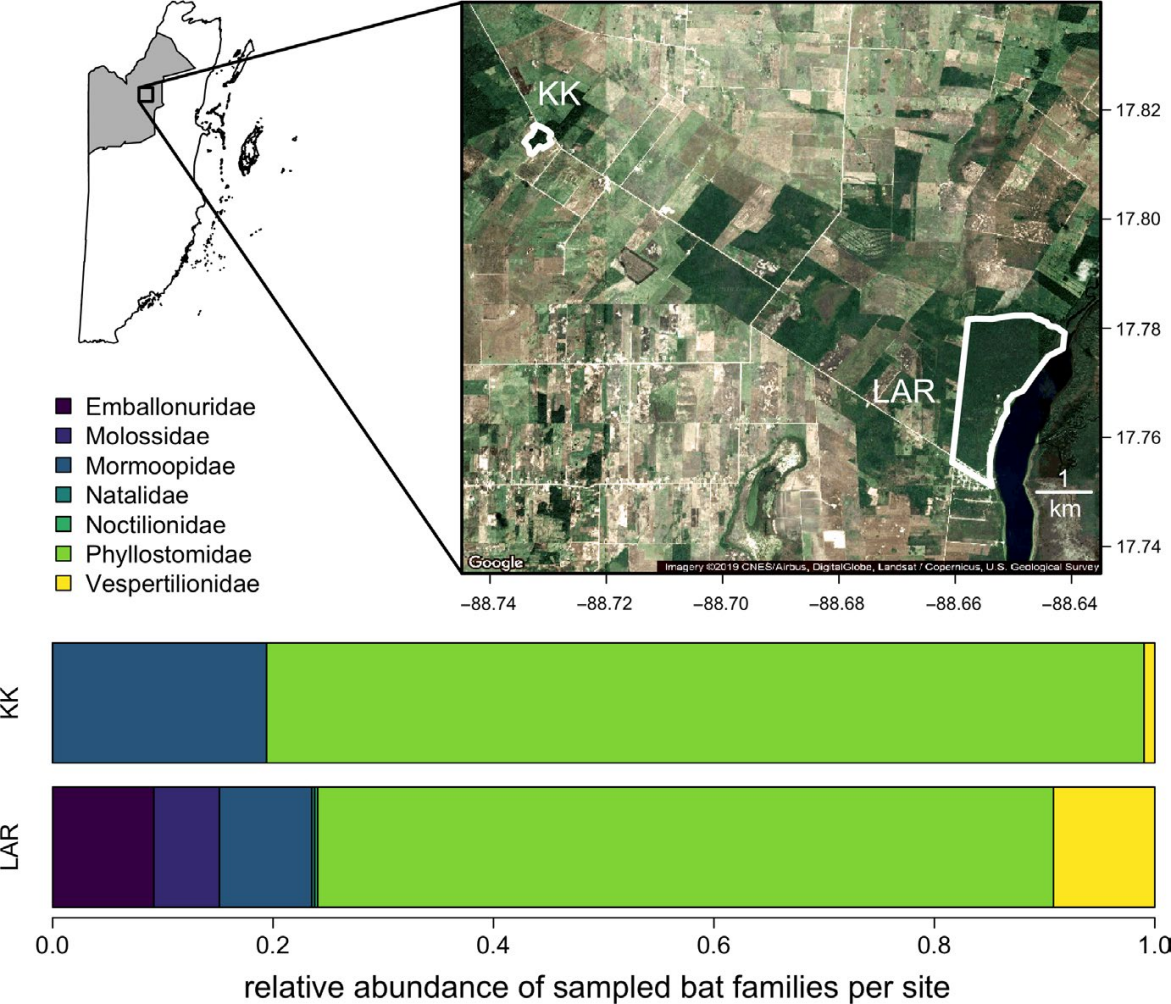
Study sites in northern Belize. The shaded inset shows the location of Orange Walk District. Borders show the boundaries of the LAR (Lamanai Archaeological Reserve) and KK (Ka’Kabish). White and brown shading indicates agricultural and urban development, while dark green shading represents intact forest. Satellite imagery was derived from Google Maps. Stacked bar plots show the relative abundance of each sampled bat family per study site [Colour figure can be viewed at wileyonlinelibrary.com]

From April 24 to May 6, 2017 and from April 23 to May 5, 2018, we sampled 469 bats from 33 species captured in two adjacent areas in the Orange Walk District of Belize: Lamanai Archaeological Reserve (LAR) and Ka’Kabish (KK; Figure 1). The LAR is bordered by the New River Lagoon, forest and agriculture, while KK is a remnant patch of forest surrounded by agriculture located 10 km away. We consider these sites to be independent, as the small home ranges of many Neotropical bat species and lack of continuous forest between LAR and KK have probably restricted most individual bat intersite movement (Jones, Hämsch, Page, Kalko, & O’mara, 2017; Loayza & Loiselle, 2008); however, some species may have greater connectivity (e.g., inter‐roost movement of *Desmodus rotundus* occurs rarely). At least 44 of the 70 bat species in Belize have been recorded in this region (Herrera et al., 2018; Reid, 1997). Bats were captured with mist nets primarily along flight paths and occasionally at the exits of roosts from 7 p.m. until 10 p.m. Harp traps were also set from 6 p.m. to 5 a.m. In KK, *D. rotundus*, *Trachops cirrhosus* and *Chrotopterus auritus* were captured at their shared roost. In the LAR, *D. rotundus*, *Saccopteryx bilineata* and *Glossophaga soricina* were sampled from a shared roost, although individuals were also sampled along flight paths across the greater site. More broadly, patterns of roost sharing of bat species in northern Belize remain elusive.

Bats were placed in cloth bags until processing and were identified to species (and sex) based on morphology (Reid, 1997). Reproductive activity was indicated by the presence of scrotal testes in males and by evidence of pregnancy or lactation in females; across bat species, 69% of males and 42% of females were in reproductive condition. We also visually screened bats for the presence of ectoparasites (i.e., bat flies, ticks, bat bugs, mites; Ter Hofstede, Fenton, & Whitaker, 2004). We collected 3–30 µl of blood (volumes were dependent on bat mass) by lancing the propatagial vein with a sterile needle. Blood was collected with heparinized capillary tubes and stored on Whatman FTA cards to preserve bacterial DNA. Field procedures followed guidelines for the safe and humane handling of bats published by of the American Society of Mammalogists (Sikes, The Animal Care and Use Committee of the American Society of Mammalogists, 2016) and were approved by the Institutional Animal Care and Use Committees of the University of Georgia (A2014 04‐016‐Y3‐A5) and American Museum of Natural History (AMNHIACUC‐20170403 and AMNHIACUC‐20180123). Fieldwork was authorized by the Belize Forest Department under permits WL/2/1/17(16), WL/2/1/17(19) and WL/2/1/18(16). Sample size was similar between years (2017 = 202, 2018 = 267) but varied by site (LAR = 365, KK = 101). More species were sampled for blood at LAR (*n* = 33) than at KK (*n* = 17; Figure 1), reflecting site differences in species richness (Herrera et al., 2018). We sampled 1–139 individuals per bat species (the maximum being *D. rotundus*), with a mean of 14 individuals per bat species (Table S1).

### 2.2 DNA extraction, PCR amplification and amplicon sequencing

Genomic DNA was extracted from blood on FTA cards using QIAamp DNA Investigator Kits (Qiagen). We tested DNA for haemoplasmas using PCR (polymerase chain reaction) with primers and procedures described in prior analyses (Volokhov, Becker, et al., 2017). We included blank FTA punches as an extraction control, ultrapure water as a negative control and *Candidatus* Mycoplasma haemozalophi DNA as a positive control (Volokhov et al., 2011). Amplicons from PCR‐positive samples were purified by electrophoresis and extracted using the QIAquick Gel Extraction Kit (Qiagen).

To determine haemoplasma infection status, all 16S rRNA amplicons were directly sequenced by Macrogen (https://www.macrogenusa.com/). Amplicons were sequenced with the same primers used for PCR amplification and then with internal (walking) primers when needed (Volokhov, Becker, et al., 2017). Negative DNA samples were tested for amplification quality using the universal PCR primers targeting the mammal mitochondrial 16S rRNA gene (Volokhov, Kong, George, Anderson, & Chizhikov, 2008) or the mitochondrial cytochrome *c* oxidase subunit 1 (COI) gene (Clare, Lim, Engstrom, Eger, & Hebert, 2007); all haemoplasma‐negative DNA samples gave positive signal in the mitochondrial 16S rRNA gene‐ and/or the COI‐specific PCR assays. All amplified sequences were subjected to chimeric sequence analysis using decipher (Wright, Yilmaz, & Noguera, 2012) and uchime (Edgar, Haas, Clemente, Quince, & Knight, 2011). All haemoplasma sequences have been deposited in GenBank under accession nos. MH245119–MH245194 and MK353807–MK353892; four positive samples were identified as *Bartonella* spp. during sequencing and were considered haemoplasma negative in our analyses.

### 2.3 Bat phylogenetic data

We used the rotl and ape packages in R to extract a bat phylogeny from the Open Tree of Life and to calculate branch lengths with Grafen's method (Michonneau, Brown, & Winter, 2016; Paradis, Claude, & Strimmer, 2004). To assess haemoplasma genotype sharing as a function of host phylogenetic similarity, we derived pairwise phylogenetic distances between the 33 sampled bat species (Figure S1). Because more evolutionarily distant and distinct species could display less frequent bacterial genotype sharing owing to ecological and immunological barriers to pathogen exposure and replication (Huang, Drake, Gittleman, & Altizer, 2015), we used the picante package and our bat phylogeny to derive evolutionary distinctiveness (Kembel et al., 2010).

### 2.4 Host species trait data

We obtained species‐level data on host traits relevant to pathogen transmission from previously published sources (Table S2). We obtained fecundity (litter size, litters per year), body mass and diet from the Amniote Life History and EltonTraits databases (Myhrvold et al., 2015; Wilman et al., 2014). For foraging ecology, which could affect bacterial exposure (e.g., trophic interactions; Kellner et al., 2018), we defined three dietary guilds: frugivory (including nectarivory; *n* = 11), insectivory (*n* = 18) and carnivory (including sanguivory and piscivory, *n* = 4; González‐Salazar, Martínez‐Meyer, & López‐Santiago, 2014). We also considered the proportion of plant‐based items in the diet. We simplified foraging strata into aerial (*n* = 14), arboreal (*n* = 16, including scansorial), and ground‐ or aquatic‐level foraging (*n* = 3). We also expanded prior compilations of wing aspect ratios and roost preferences to serve as proxies for ecological overlap among species (Fenton et al., 2001; Herrera et al., 2018; Reid, 1997). Roost type was simplified to open (e.g., only foliage; *n* = 6) or closed (e.g., hollows, caves; *n* = 27), and roost flexibility was simplified to using one (*n* = 16) or multiple roost types (*n* = 17). We classified maximum colony sizes as small‐to‐medium (e.g., <100 individuals; *n* = 20) or large (e.g., hundreds to thousands; *n* = 13; Reid, 1997; Santana, Dial, Eiting, & Alfaro, 2011), as most values were reported in ranges. We did not record pairwise sympatry (e.g., Luis et al., 2015; McKee et al., 2019) given that all species occur in Belize (Figure S2). Yet because more widely distributed species could have more opportunities for pathogen sharing due to range overlap, we used the geosphere package and data from the International Union for Conservation of Nature to derive geographical range size (Baillie, Hilton‐Taylor, & Stuart, 2004; Hijmans, Williams, Vennes, & Hijmans, 2019). Missing species‐level traits were taken from other databases, primary literature or closely related species (Table S2).

### 2.5 Individual‐level analysis of bat infection status

We first used the prevalence package to estimate haemoplasma infection prevalence and its 95% confidence interval (CI; Wilson method). We then used phylogenetic generalized linear mixed models (GLMMs) to test if infection status varied by sex, reproductive status, year, site and ectoparasite presence while accounting for bat phylogenetic relatedness. We fit candidate GLMMs that considered all fixed effects as well as interactions between sex and reproduction and between site and year. We also considered a model that excluded site to account for possible nonindependence of LAR and KK alongside an intercept‐only model. As vampire bats were banded for a mark–recapture study (Volokhov, Becker, et al., 2017) and some were sampled between and within years (*n* = 14), we randomly selected one of each recapture. After removing recaptures and missing values (*n* = 323), we fit the phylogenetic GLMMs using the brms package, default priors, and infection status as a Bernoulli‐distributed response. We included random effects for bat species and phylogeny, the latter of which used the phylogenetic covariance matrix (Bürkner, 2017). We ran four chains for 20,000 iterations with a burn‐in period of 10,000, thinned every 10 steps, for a total of 4,000 samples. We compared GLMMs using the leave‐one‐out cross‐validation (LOOIC) and assessed fit with a Bayesian *R* ^2^, including the total modelled variance and that attributed to only the fixed effects (Gelman, Goodrich, Gabry, & Vehtari, 2019; Vehtari, Gelman, & Gabry, 2017). We then estimated fixed effects (means and 95% highest density intervals [HDIs]) from the posterior distributions of each predictor from the top GLMM. Lastly, we assessed the sensitivity of individual‐level analyses to over‐representation of *D. rotundus* by randomly subsampling this species by the maximum *n* for other bat species.

### 2.6 Species‐level analysis of haemoplasma prevalence

We next calculated infection prevalence per species, using the metafor package to estimate logit‐transformed proportions and sampling variances (Viechtbauer, 2010). We used the nlme package to estimate phylogenetic signal as Pagel's λ with a weighted phylogenetic generalized least squares (PGLS) model to account for within‐species variance (Garamszegi, 2014). We next used a graph‐partitioning algorithm, phylogenetic factorization, to flexibly identify clades with significantly different prevalence estimates at various taxonomic depths. We used the taxize package to obtain a bat taxonomy from the National Center for Biotechnology Information (Chamberlain & Szöcs, 2013) and used the phylofactor package to partition prevalence as a Bernoulli‐distributed response in a GLM (Washburne et al., 2019). We determined the number of significant bat clades using Holm's sequentially rejective test with a 5% family‐wise error rate.

To identify species trait correlates of prevalence, we fit 11 PGLS models (weighted by sampling variance) with body mass, annual fecundity (litters per year × pups per litter), dietary guild, quantitative diet, foraging strata, aspect ratio, roost type, roost flexibility, colony size, geographical range size and evolutionary distinctiveness as predictors. We also fit PGLS models with only sample size or an intercept. We compared models with Akaike's information criterion corrected for small sample sizes (AICc) and estimated *R* ^2^ (Burnham & Anderson, 2002).

### 2.7 Hemoplasma phylogenetic analyses and genotype assignment

We compared our 16S rRNA gene sequences to those in GenBank (Volokhov, Becker, et al., 2017; Volokhov et al., 2011). Briefly, we aligned sequences using clustal x, and inter‐ and intraspecies similarity values were generated using bioedit. Genetic distances were calculated with the Kimura 2‐parameter and Tamura–Nei models, and the phylogeny was constructed using mega x with the minimum evolution algorithm (Kumar, Stecher, Li, Knyaz, & Tamura, 2018).

We assigned haemoplasma genotypes to positive bats based on analysis of the 16S rRNA partial gene (860–1,000 bp) sequences in GenBank and their clustering on the phylogeny. Genotypes were designated as novel if (a) sequences differed from the closest haemoplasma sequences in GenBank by ≥1.5% and/or (b) if sequence similarity was <1.5% but genotype‐specific reproducible mutations (at least two per sequence) were observed between haemoplasma sequences from at least two independent bat samples and the nearest GenBank haemoplasma sequences. These genotype‐specific mutations were further used to differentiate closely related haemoplasma genotypes from our sample. We caution that genotype is not synonymous with species, as analysis of the 16S rRNA gene alone is insufficient for accurate species identification of *Mycoplasma* spp. (Volokhov et al., 2012). Future studies using genomics or housekeeping genes may identify independent but closely related haemoplasma species in our genotypes.

To assess if haemoplasma genotype assignments were associated with site and year, we used χ^2^ tests with *p* values generated through a Monte Carlo procedure. Prior to our phylogenetic and network analyses of genotype distributions across bat species (see below), we used another χ^2^ test to assess the association between haemoplasma genotype identify and bat host identity.

### 2.8 Evolutionary relationships between bats and haemoplasmas

To determine the degree to which bat haemoplasma genotypes display host specificity and to describe their evolutionary relationships with host species, we used our bat and haemoplasma phylogenies to construct a binary association matrix. To test the dependence of the haemoplasma phylogeny upon the bat phylogeny and thus assess evidence of evolutionary codivergence, we applied the Procrustes Approach to Cophylogeny (PACo) using distance matrices and the paco package (Hutchinson, Cagua, Balbuena, Stouffer, & Poisot, 2017). We used a jackknife procedure to estimate the degree to which each bat–genotype link supported a hypothesis of phylogenetic congruence; links were supported if their upper 95% confidence interval was below the mean of all squared jackknife residuals (Balbuena, Míguez‐Lozano, & Blasco‐Costa, 2013).

### 2.9 Haemoplasma genotype sharing among bat species

We used haemoplasma genotype assignments to create a network, with each node representing a bat species and edges representing shared genotypes among bat species pairs. We built an adjacency matrix using the igraph package and used the Louvain method to assess the structure of bat–haemoplasma communities within this network (Csardi & Nepusz, 2006). To test whether the distribution of haemoplasma genotypes across our Neotropical bat species is shaped by host phylogeny, we used two GLMs to predict counts of shared genotypes (Poisson errors) and the presence of sharing (binomial errors) by phylogenetic distance between bat species. We assessed statistical significance with a quadratic assignment procedure via the sna package (Butts, 2008).

We calculated two metrics of network centrality to quantify different aspects of how important a node (bat species) is to haemoplasma genotype sharing: degree and eigenvector centrality (Bell, Atkinson, & Carlson, 1999). Whereas degree indicates the number of other species with which a host shares bacterial genotypes (i.e., links per node), eigenvector centrality indicates the tendency for a host to share genotypes with species that also share more genotypes (i.e., connectivity). Eigenvector centrality is thus an extension of degree that can identify hubs of parasite sharing (Gómez, Nunn, & Verdú, 2013). These two metrics were moderately correlated (*ρ* = 0.59), with many non‐zero degree species displaying zero eigenvector centrality. To examine spatial and temporal patterns in host centrality, we built separate adjacency networks per each site and year. We fit separate GLMs to determine how haemoplasma sharing centrality was predicted by site, year and the two‐way interaction. Degree was modelled as a Poisson‐distributed response, while eigenvector centrality was logit‐transformed and used Gaussian errors. We next applied phylogenetic factorization to both metrics and weighted the algorithms by the square‐root sample size per species (Garamszegi, 2014). We then fit the same PGLS models used in our prevalence analysis to identify the most competitive trait predictors of bat species centrality to haemoplasma sharing. Lastly, to assess whether network centrality is associated with haemoplasma prevalence, we fit two weighted PGLS models with each centrality metric as a univariate predictor.

## 3 RESULTS

### 3.1 Haemoplasma infection status

**Figure 2:**
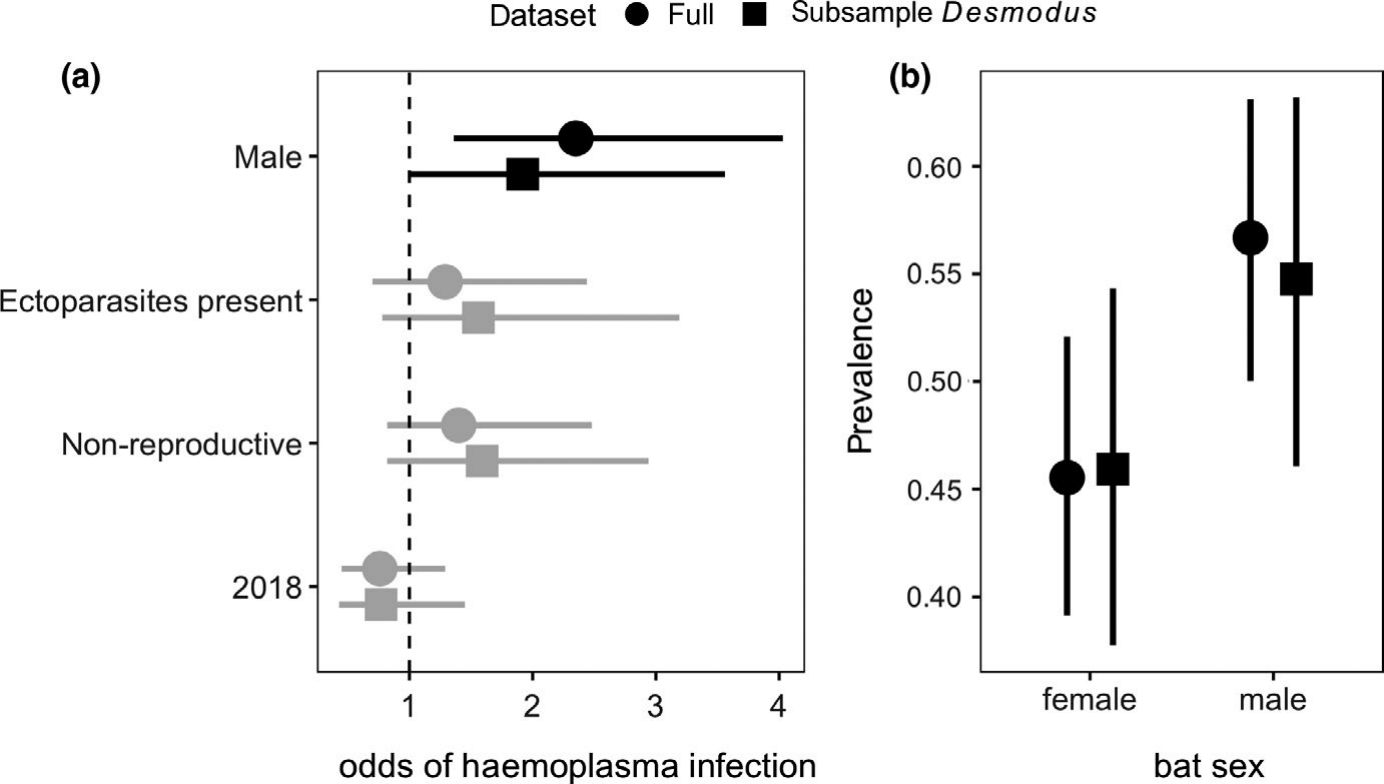
Predictors of individual bat haemoplasma infection status. (a) Odds ratios and 95% HDIs from the most parsimonious phylogenetic GLMM (Table S4). Estimates that do not overlap with 1 (dashed line) are displayed in black. Reference levels for the odds ratios include bats sampled at LAR, females, reproductive bats, absence of ectoparasites and bats sampled in 2017. (b) Infection prevalence and 95% confidence intervals (Wilson method) stratified by sex. Results are shown for the full data set and after randomly subsampling *Desmodus rotundus*

We detected sequence‐confirmed haemoplasma infection in 239 of 469 individuals (51%; 95% CI: 46%–55%), with positive individuals in 23 of the 33 sampled bat species (Table S3). The most parsimonious phylogenetic GLMM explained 22% of the modelled variance in infection status and included only sex, reproductive status, ectoparasites and year (ΔLOOIC = 0.16, *w_i_* = .27; Table S4), suggesting site to be uninformative. Males had higher odds of infection than females (odds ratio [OR] = 2.35, 95% HDI: 1.36–4.03), but risk was unrelated to ectoparasitism, reproduction or year (Figure 2a). This result was weakly sensitive to over‐representation by *Desmodus rotundus*, as effect size and the prevalence sex difference was weaker when we randomly subsampled this species (Figure 2). Across all models, fixed effects only explained up to 7% of the modelled variance, suggesting more variation explained by the species and phylogeny random effects.

### 3.2 Interspecies variation in haemoplasma prevalence

**Figure 3:**
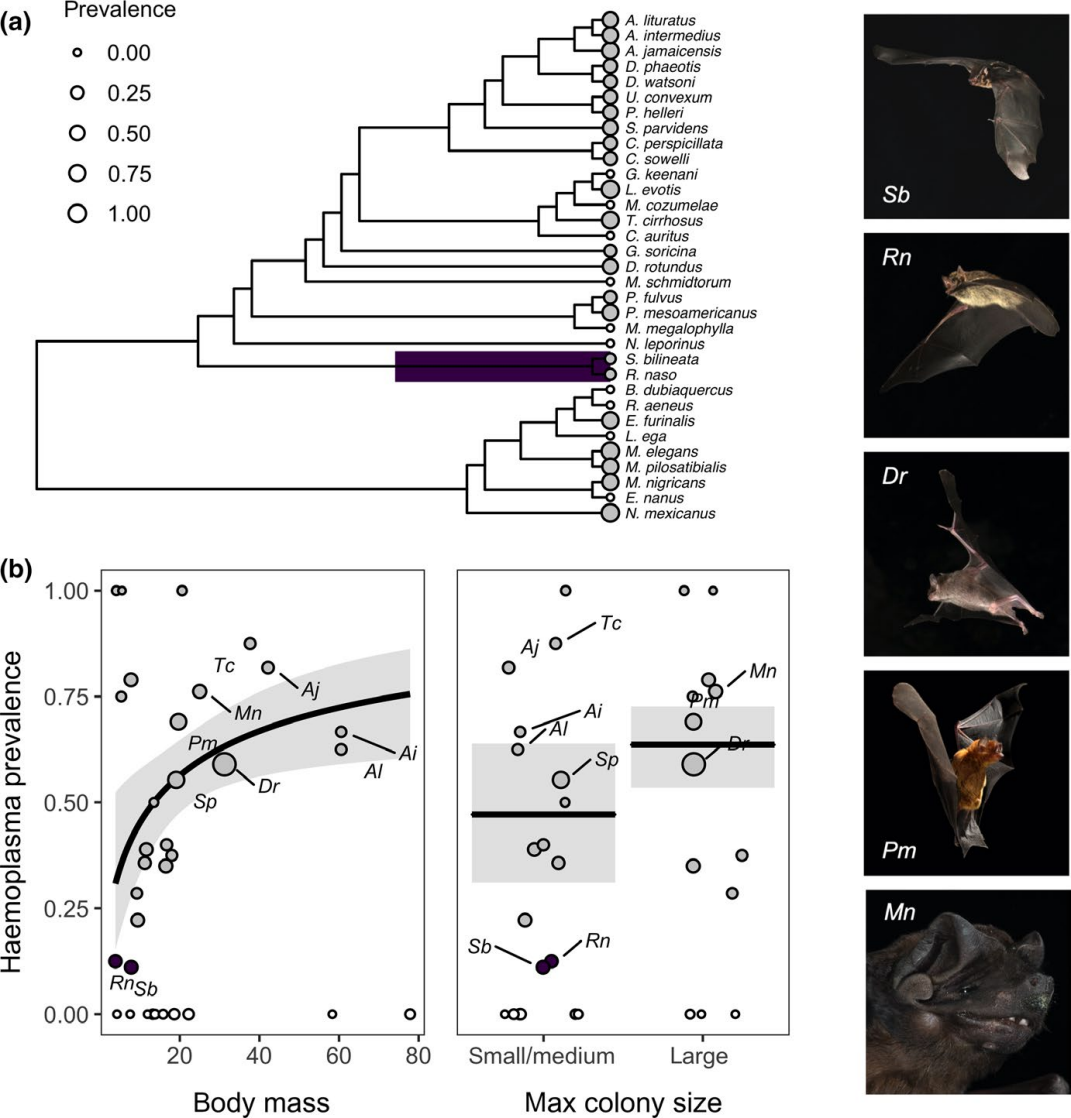
Predictors of species‐level haemoplasma prevalence across the Belize bat community. (a) Clades with significantly different prevalence are highlighted. (b) Results from the top PGLS models predicting prevalence as a function of mass and colony size. Model fit and 95% confidence intervals are shown overlaid with data scaled by sample size; species from the clade identified through phylogenetic factorization are coloured as in (a). Species identified by phylogenetic factorization (*Saccopteryx bilineata* and *Rhynchonycteris naso*) and with larger body mass, colony size and haemoplasma prevalence (*Desmodus rotundus*, *Molossus nigricans* and *Pteronotus mesoamericanus*) are shown to the right (photographs by Sherri and Brock Fenton) [Colour figure can be viewed at wileyonlinelibrary.com]

Across bat species, haemoplasma prevalence ranged from 0% to 100% (mean = 37%). We estimated Pagel's λ in logit‐transformed prevalence to be .39, indicating moderate phylogenetic signal. Similarly, phylogenetic factorization identified one bat clade with significantly lower prevalence compared to the paraphyletic remainder: the Emballonuridae (12% infected; Figure 3a). Our trait‐based analysis showed that relatively larger species (*β* = 1.48, *p* = .01, *R* ^2^ = .24) and those with larger colonies (*β* = 0.67, *p* = .06, *R* ^2^ = .20) had higher prevalence (Figure 3b; Table 1). Relatively heavier (≥20 g) and larger colony species included *D. rotundus*, *Molossus nigricans* and *Pteronotus mesoamericanus*, for which prevalence was greater than 58%. Although these three species were also heavily sampled, other well‐sampled species such as *Sturnira parvidens* and *Carollia sowelli* had lower prevalence, and sample size did not predict prevalence (Table 1).

**Table 1:** Competing weighted phylogenetic generalized least squares models predicting haemoplasma infection prevalence (logit‐transformed) across the Belize bat community

Model structure	*k*	ΔAICc	*w_i_*	*R* ^2^
Log body mass	2	0.00	.40	.24
Maximum colony size	2	1.71	.17	.20
Roost type	2	2.82	.10	.18
Foraging strata	3	2.88	.09	.24
Roost flexibility	2	3.56	.07	.16
Per cent plants in diet	2	4.30	.05	.14
Dietary guild	3	5.46	.03	.17
Log evolutionary distinctiveness	2	5.50	.02	.11
Log aspect ratio	2	5.61	.02	.10
Log annual fecundity	2	6.13	.02	.09
Square‐root geographical range size	2	6.27	.02	.08
1 (intercept only)	1	6.77	.01	.00
Sample size	2	8.38	.01	.02

Note

Models are ranked by ΔAICc with the number of coefficients (*k*), Akaike weights (*w_i_*) and a likelihood ratio test pseudo‐*R* ^2^.

### 3.3 Hemoplasma genotype diversity

Our phylogenetic analysis identified 29 *Mycoplasma* genotypes in the Belize bat community (Table 2), including three previously identified from vampire bats (VBG1–3; Volokhov, Becker, et al., 2017). All genotypes demonstrated minor levels of sequence variability (99.5%–100%; Table 2). Based on comparisons with sequences from GenBank, 21 of these genotypes represent novel haemoplasmas, five are closely related to nonhaemoplasma mycoplasmas, and many are phylogenetically related to previously identified *Mycoplasma* spp. and haemoplasmas identified from other bat species (e.g., Di Cataldo et al., 2020; Millán et al., 2019; Millán, López‐Roig, Delicado, Serra‐Cobo, & Esperón, 2015), bat ticks (e.g., Hornok et al., 2019), primates including humans (e.g., Alcorn et al., 2020; Bonato, Figueiredo, Gonçalves, Machado, & André, 2015; Hattori et al., 2020) and rodents (e.g., Gonçalves et al., 2015; Goto, Yasuda, Hayashimoto, & Ebukuro, 2010; Vieira et al., 2009). A more detailed description of these 29 bacterial genotypes is provided in the Figure S3.

**Table 2:** Haemoplasma genotypes identified from the Belize bat community. Genotypes are given with their bat host species, representative GenBank numbers and intragenotype variability

Genotype	Host species	Representative GenBank number	Mean intragenotype sequence similarity (%)
VBG1	*Desmodus rotundus*, *Pteronotus fulvus* ^c^	KY932701	99.8
VBG2	*Desmodus rotundus*	KY932678	99.9
VBG3	*Desmodus rotundus*	KY932722	99.6
CS1^b^	*Carollia sowelli*	MK353833	100
CS2^b^	*Carollia sowelli*, *C. perspicillata*	MH245134	99.7
MR1^b^	*Molossus nigricans*	MH245174	99.7
MR2^b^	*Molossus nigricans*	MH245151	NA^a^
PPM^b^	*Pteronotus mesoamericanus*, *P. fulvus* ^c^	MH245159	99.9
EF1^b^	*Eptesicus furinalis*, *Saccopteryx bilineata* ^c^, *Glossophaga soricina* ^c^	MH245147	99.6
EF2^b^	*Eptesicus furinalis*	MH245131	99.9
NM^b^	*Natalus mexicanus* ^c^	MK353818	NA^a^
LE^b^	*Lophostoma evotis*	MK353892	99.9
TC1^b^	*Trachops cirrhosus*	MH245145	99.8
TC2^b^	*Trachops cirrhosus*	MK353860	99.8
APH1^b^	*Dermanura phaeotis*, *D. watsoni*, *A. lituratus* ^c^	MH245132	100
APH2^b^	*Artibeus jamaicensis*, *A. lituratus*, *A. intermedius*	MH245187	99.9
APH3^b^	*Artibeus intermedius*	MH245186	99.8
GLS^b^	*Glossophaga soricina*	MK353874	99.5
MYE^b^	*Myotis elegans*, *Myotis pilosatibialis* ^c^	MK353840	100
MYK^b^	*Myotis pilosatibialis* ^c^	MH245153	NA^a^
UB^b^	*Uroderma convexum*	MK353869	99.8
PLU^b^	*Platyrrhinus helleri* ^c^, *Uroderma convexum* ^c^	MK353883	99.6
SP^b^	*Sturnira parvidens*; *A. lituratus* ^c^	MH245168	99.4
RHN^b^	*Rhynchonycteris naso* ^c^	MK353871	NA^a^
*M. moatsii*‐like 1^d^	*Pteronotus mesoamericanus* ^c^	MK353864	NA^a^
*M. moatsii*‐like 2^d^	*Myotis pilosatibialis* ^c^	MK353862	NA^a^
*M. moatsii*‐like 3^d^	*Rhynchonycteris naso* ^c^	MH245146	NA^a^
*M. lagogenitalium*‐like^d^	*Glossophaga soricina* ^c^	MH245140	NA^a^
*M. muris*‐like^d^	*Saccopteryx bilineata* ^c^	MH245138	NA^a^

^a^ Intragenotype sequence variability could not be assessed, as only one sequence was identified.

^b^ Novel haemoplasma genotypes.

^c^ Genotypes were detected in only one individual of these bat species.

^d^ Non‐haemoplasma *Mycoplasma* genotypes.

After controlling for multiple comparisons, our 29 bacterial genotypes were associated with site (χ^2^ = 47.11, *p* < .01) and year (χ^2^ = 40.40, *p* < .01). Genotype composition was more diverse at LAR (Figure S4), and KK haemoplasmas were dominated by vampire bat genotypes (VBG1–3). Genotype composition was more idiosyncratic by study year. However, these 29 bacterial genotypes were most strongly associated with bat species (χ^2^ = 3,532, *p* < .01; Figure S4).

### 3.4 Bat–haemoplasma evolutionary relationships

**Figure 4:**
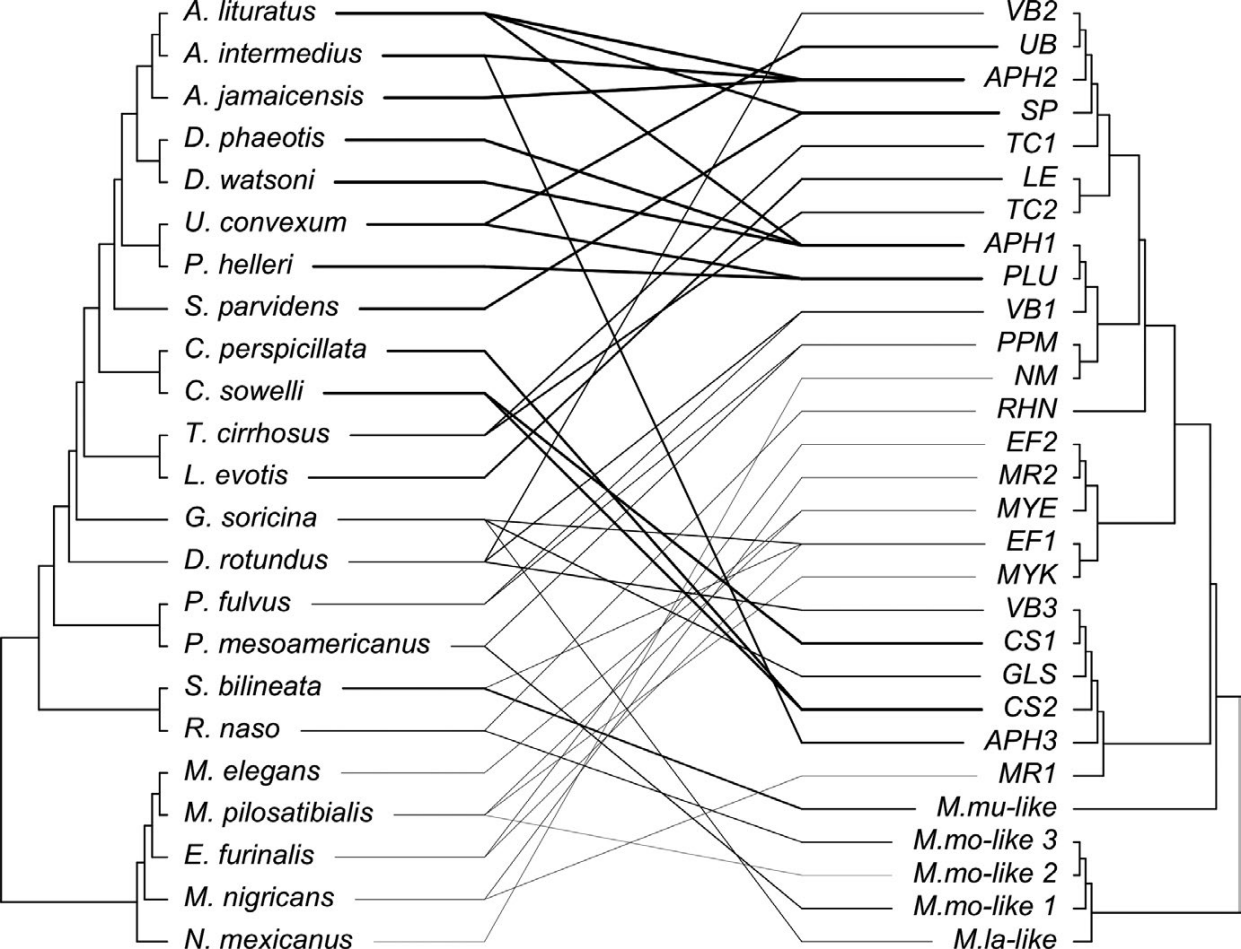
Evolutionary relationships between Belize bats and haemoplasma genotypes. The cophylogeny plot shows the bat phylogeny on the left and the haemoplasma genotype phylogeny on the right. We used the treespace package to collapse our complete haemoplasma phylogeny (Figure S3) to only the 29 bacterial genotypes (Jombart, Kendall, Almagro‐Garcia, & Colijn, 2017). Lines display bat–haemoplasma associations and are shaded by the inverse of the squared residuals from PACo (i.e., dark lines show small residuals more indicative of coevolution)

Although some haemoplasma genotypes were shared between bat species (i.e., VBG1, CS2, PPM, EF1, AH1–2, MYE, PLU, SP; *n* = 9), most showed strong host specificity (*n* = 20; Table 2). Our coevolutionary analysis (PACo) supported congruence between the bat and haemoplasma phylogenies ( $m_{\mathrm{XY}}^{2}$ = 36.21, *p* = .02, *n* = 1,000; Figure 4), suggesting that haemoplasma evolution has mostly tracked bat speciation. However, PACo also demonstrated that only 56% of the 41 unique bat–haemoplasma links displayed significant evidence of coevolution (Figure S5), and these patterns were almost exclusively found within the Phyllostomidae (with the exception of *Saccopteryx bilineata* and its *Mycoplasma muris*‐like bacterial genotype). The other 18 bat–haemoplasma links therefore displayed evidence of phylogenetic incongruence and thus probable host shifts.

### 3.5 Hemoplasma genotype sharing networks

**Figure 5:**
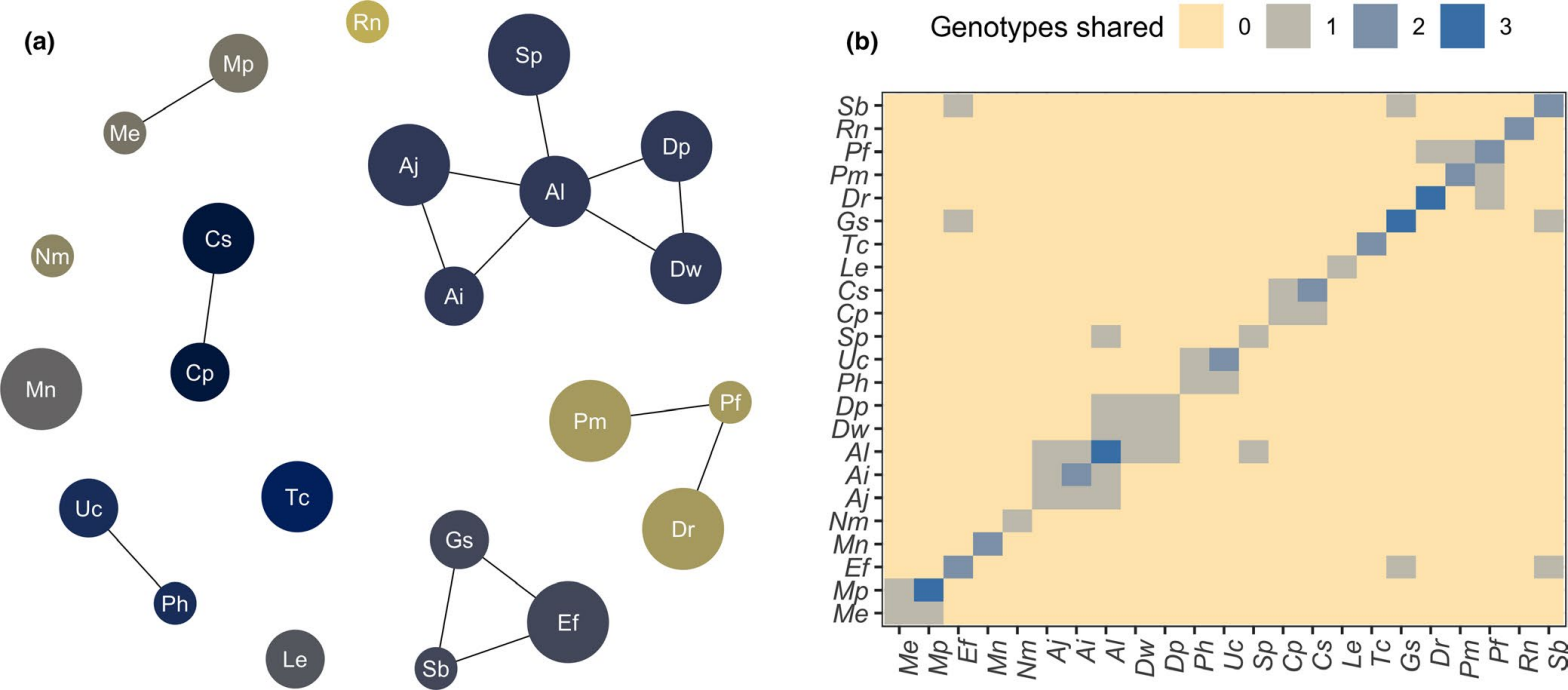
Patterns of haemoplasma genotype sharing across the Belize bat community. (a) Nodes in the genotype network represent bat species (abbreviated by Latin binomials), and edges represent a shared genotype. Nodes are coloured by communities identified with the Louvain method and are scaled by the number of individuals per species. (b) Matrix showing pairwise haemoplasma genotype sharing, coloured by the number of genotypes shared between bat species [Colour figure can be viewed at wileyonlinelibrary.com]

Within our bat–haemoplasma network, genotype sharing was restricted to five host communities, whereas six genotypes were each restricted to a single bat species (Figure 5a). GLMs showed that both the frequency and the presence of genotype sharing declined with phylogenetic distance between bat species (Poisson: *p* < .001, *R* ^2^ = .08; binomial: *p* < .001, *R* ^2^ = .51; Figure 5b).

Bats shared haemoplasma genotypes with zero to five other species (i.e., degree), and most hosts had eigenvector centrality of zero (Figure S6). Six bat species had nonzero eigenvector centrality (37%–100%), indicating that they shared more haemoplasma genotypes with other highly connected host species. Stratifying our haemoplasma network across sites and years showed that centrality measures varied by space but not time (Figure S7; Table S5). We observed no haemoplasma sharing at KK, which probably reflects lower host diversity (Herrera et al., 2018).

**Figure 6:**
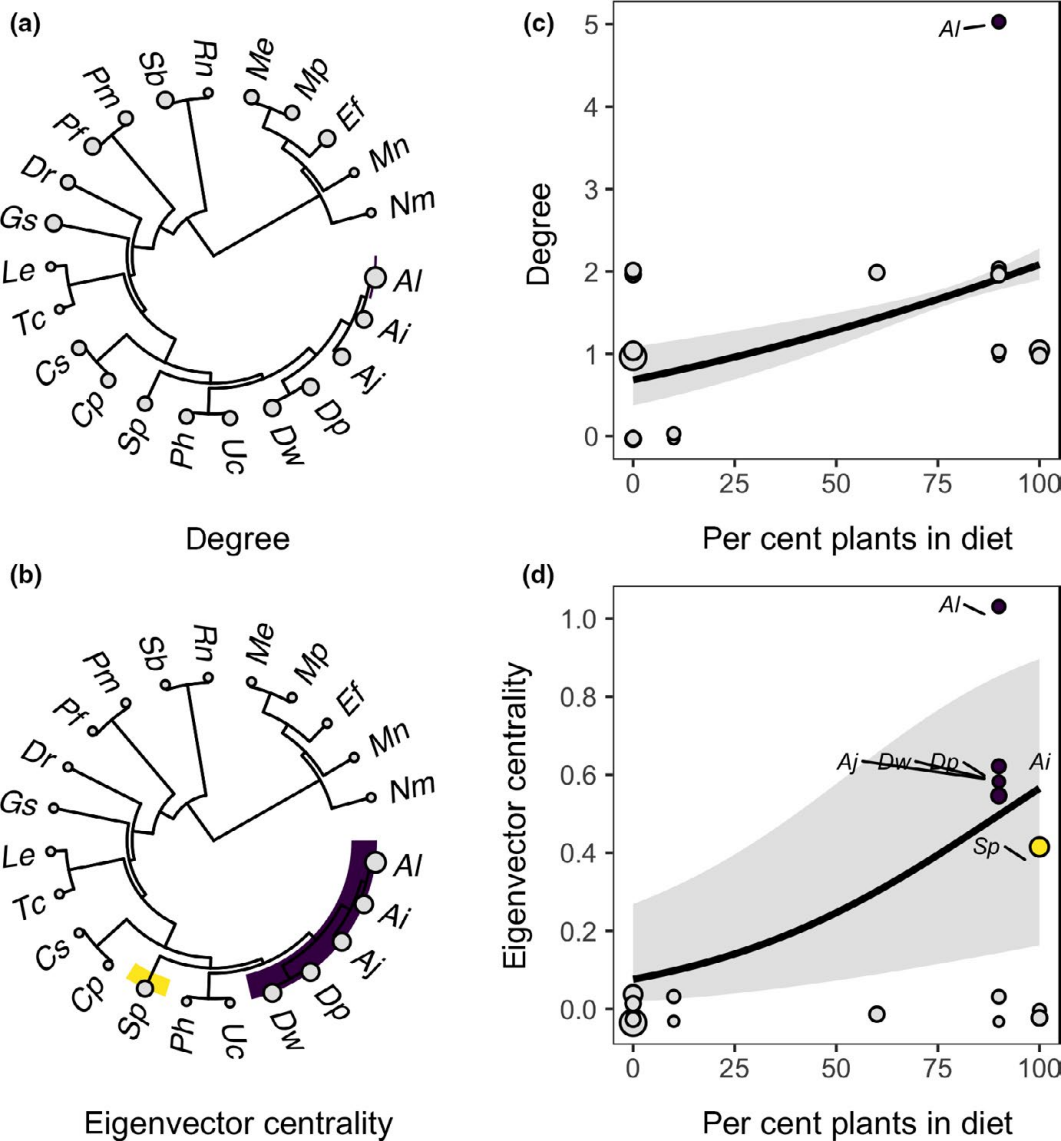
Phylogenetic patterns in haemoplasma genotype networks for Belize bat species (a) degree and (b) eigenvector centrality. Clades showing significantly different centrality metrics are highlighted, and points are scaled by observed values. Results from the top PGLS models predicting both centrality metrics as a function of bat species traits (c and d). Model fit and 95% confidence intervals are shown overlaid with data scaled by sample size; species from the clades identified through phylogenetic factorization are coloured as in (a) and (b) [Colour figure can be viewed at wileyonlinelibrary.com]

Phylogenetic factorization identified similar bat clades with significantly different centrality compared to the paraphyletic remainder (Figure 6a, b). For degree, the algorithm only identified *Artibeus lituratus* as being more central ( $\bar{x}$ = 5) than other bats ( $\bar{x}$ = 1.14). However, phylogenetic factorization identified three taxa in the subfamily Stenodermatinae that had significantly elevated eigenvector centrality: the genera *Artibeus* and *Dermanura* ( $\bar{x}$ = .67 compared to $\bar{x}$ = .02 in all other bats), the species *A. lituratus* ( $\bar{x}$ = 1 compared to $\bar{x}$ = .12), and the species *S. parvidens* ( $\bar{x}$ = .37 compared to $\bar{x}$ = .15). Mirroring these results, phylogenetic signal was absent for degree (λ = 0) but high for eigenvector centrality (λ = .93).

Trait‐based analyses showed that degree centrality was best predicted by diet (Table S6); bat species feeding more heavily on fruit and nectar shared more bacterial genotypes with other species (*β* = 0.004, *p* < .001, *R* ^2^ = .20; Figure 6c). Similarly, eigenvector centrality was best predicted by bat colony size and diet (Table S7); highly central species had small colonies (*β*_large_ = –1.93, *p* = .05, *R* ^2^ = .13) and fed more on plants (*β* = 0.03, *p* < .01, *R* ^2^ = .10; Figure 6d).

As a final analysis, we assessed whether network centrality (i.e., a bat species’ role in haemoplasma genotype sharing) predicted contemporary infection prevalence (Figure S8). However, we found no associations between species‐level infection prevalence and centrality as measured by degree (*β* = –0.13, *R* ^2^ = .03, *p* = .42) or eigenvector centrality (*β* = –0.20, *R* ^2^ < .01, *p* = .79).

## 4 DISCUSSION

By examining the prevalence and distribution of a common bacterial pathogen (haemoplasmas) in a diverse bat community, we expanded analysis of the ecological and evolutionary predictors of bat infection and pathogen sharing beyond viruses. Across the bat community, haemoplasma infection risk was weakly higher for males but was better predicted by phylogeny, with large‐bodied and large‐colony bat species showing greater prevalence. Haemoplasmas showed high diversity and mostly strict host associations, with strong congruence between the bat and haemoplasma phylogenies. Although codivergence was supported by our analyses, we also observed haemoplasma genotype sharing and evidence of historical host shifts between closely related bats. Species most central to this haemoplasma sharing network displayed taxonomic clustering and were disproportionately frugivores and nectarivores. Yet these highly central bat species did not also have the highest haemoplasma prevalence, reinforcing mostly infrequent bacterial sharing between species. Our work reveals phylogenetic patterns in haemoplasma infection in a diverse bat community while contributing to broader efforts to understand the host specificity of bacterial pathogens and their cross‐species transmission patterns in wildlife.

Whereas many bacterial pathogens, including haemoplasmas, are common in bats (Bai et al., 2011; Becker et al., 2018; Ikeda et al., 2017; Mascarelli et al., 2014; Millán et al., 2015; Volokhov, Becker, et al., 2017), the factors that confer high infection probability are poorly understood. In the Belize bat community, the odds of haemoplasma infection tended to be higher in males, suggesting male‐biased transmission as detected in feline and canine systems (Soto et al., 2017; Walker Vergara et al., 2016). Such patterns could stem from males mounting weaker immune responses than females (Kelly, Stoehr, Nunn, Smyth, & Prokop, 2018) or to male defence of multifemale roosts in many Neotropical bat species (Voigt, von Helversen, Michener, & Kunz, 2001). Direct transmission of haemoplasmas has been demonstrated in feline and rodent systems (Cohen et al., 2018; Museux et al., 2009) but only inferred in bats from metagenomic studies detecting these bacteria in saliva (Volokhov, Becker, et al., 2017). We found weak support for the hypothesis that ectoparasites play a role in infection risks (Hornok et al., 2019; Willi, Boretti, Meli, et al., 2007). The weak male bias in infection could cast further doubt on vector‐borne transmission, as females in some bat species have elevated ectoparasitism (Frank, Mendenhall, Judson, Daily, & Hadly, 2016); however, a secondary GLMM testing this hypothesis found generally weak support for a female bias in ectoparasitism in our system (Figure S9). Future work assessing ectoparasite burdens could better elucidate the roles of vectors in haemoplasma risk.

Across Neotropical bats sampled in Belize, we found phylogeny to be a better predictor of haemoplasma risk than individual traits, site or year. Phylogenetic factorization identified one clade, the Emballonuridae, with significantly lower prevalence than all other bats in the community. This moderate phylogenetic signal mirrors comparable effects of phylogeny for bat viruses (Guy, Thiagavel, Mideo, & Ratcliffe, 2019), similarly suggesting potential for innate differences in species susceptibility or pathogen exposure. Trait‐based analyses revealed that this taxonomic pattern was driven by heavier and large‐colony species having greater haemoplasma prevalence. Small‐bodied species could have low prevalence due to small blood volumes and low bacterial titres (Volokhov, Becker, et al., 2017). Alternatively, the positive, saturating relationship between body mass and bacterial prevalence could be driven by allometric patterns in competence (Downs et al., 2019), in contrast to weak or opposite relationships between mass and viral richness across bats (Guy et al., 2019; Han, Schmidt, et al., 2016). As larger‐bodied bat species can also be more abundant in Neotropical habitat fragments (Herrera et al., 2018), these results suggest land conversion could increase the frequency of bat species most capable of maintaining haemoplasma infection. Similarly, positive relationships between colony size and prevalence could support density‐dependent transmission of bacteria (McCallum et al., 2001), whereas mixed support has been found for bat viruses (Streicker et al., 2012; Webber, Fletcher, & Willis, 2017). Future work could test how community‐wide infection patterns vary across broader habitat gradients and use multiple bacteria to assess the generality of these trends.

Approximately two‐thirds of the Neotropical bat species sampled in Belize were infected by haemoplasmas, for which we observed high genetic diversity consistent with other studies of this pathogen in bats (Mascarelli et al., 2014; Millán et al., 2015; Volokhov, Becker, et al., 2017). However, these bacterial genotypes were mostly novel and only weakly related to haemoplasmas described elsewhere in Latin America (Ikeda et al., 2017; Millán et al., 2019), with the exception of those previously identified from vampire bats (Volokhov, Becker, et al., 2017). When considering the phylogenetic scale of genotypes, most haemoplasmas were host‐specific. Over half of our haemoplasma communities consisted of a single bat–genotype association, matching the degree of host specificity observed more generally for *Mycoplasma* spp. (Citti & Blanchard, 2013; Pitcher & Nicholas, 2005). When we did detect genotype sharing between species, this occurred mostly between closely related hosts (e.g., genotype PPM was detected in *Pteronotus mesoamericanus* and *P. fulvus*), indicating bat phylogenetic distance decreased the probability of bacterial transfer.

Analyses to characterize species centrality to the haemoplasma genotype sharing network showed that one species (*Artibeus lituratus*) and the subfamily Stenodermatinae played key roles. This clade, and especially the genera *Artibeus* and *Dermanura* (formerly all classified in *Artibeus*), was the only taxon with nonzero connectivity, and this pattern was reflected in fruit‐ and nectar‐based diets and small colonies being the primary predictors of centrality. The strictly frugivorous Stenodermatinae represents a recent divergence in the Phyllostomidae (Botero‐Castro et al., 2013), and high centrality of these species may indicate weaker phylogenetic barriers for bacterial transmission between hosts in this clade. Two other analyses reinforced infrequent and conserved haemoplasma sharing between species. First, phylogenetic patterns in prevalence were distinct from those in genotype sharing centrality (e.g., large‐colony species had higher prevalence but lower connectivity), and prevalence accordingly did not predict centrality. Second, we found general congruence between bat and haemoplasma phylogenies. Although this shows codivergence is a strong evolutionary force, congruence can also stem from preferential jumps to closely related hosts (De Vienne et al., 2013). Although we cannot rule out that some host shifts may be artefacts of the limited resolution of both the phylogenies, our evolutionary analyses and genotype sharing results imply that haemoplasma host shifts are possible yet rare.

By sampling a diverse assemblage of bacterial genotypes in an ecologically and evolutionary rich host community, our work has broader implications for our understanding of disease emergence. Many bacterial pathogens are thought to be generalists and relatively unlikely to specialize in a novel host (Pedersen, Altizer, Poss, Cunningham, & Nunn, 2005; Woolhouse & Gowtage‐Sequeria, 2005), in contrast to many viruses in which host shifts are more common owing to high mutation rates and short infectious periods (Geoghegan et al., 2017; Longdon, Brockhurst, Russell, Welch, & Jiggins, 2014). Recent theoretical work suggests host shift speciation may be less common for bacteria because of higher phenotypic plasticity (e.g., the ability to reside in diverse habitats) and a slower tempo of evolution (Bonneaud et al., 2019). Obligate reliance of *Mycoplasma* spp. on host cells and more chronic infections probably explains their propensity to specialize (Citti & Blanchard, 2013; Cohen et al., 2018). More broadly, however, using genetics to infer pathogen sharing, rather than coarser phylogenetic scales (e.g., species complexes or genera), is increasingly showing that many bacterial strains may be more host‐specific (Withenshaw, Devevey, Pedersen, & Fenton, 2016). The high specialism of bat haemoplasma genotypes thus underlines the importance of using finer phylogenetic scales in the study of infectious disease (Fountain‐Jones et al., 2018; Graham, Storch, & Machac, 2018).

Comparative analyses of viruses have suggested that phylogenetically conserved pathogen jumps between species may be a broader generality in the study of disease emergence (Albery, Eskew, Ross, & Olival, 2019; Luis et al., 2015; Streicker et al., 2010). With few exceptions, our results on haemoplasma genotype sharing between Neotropical bat species are generally consistent with this pattern for a bacterial pathogen. Two cases in which haemoplasmas were shared between more distantly related species included the VBG1 genotype in *Desmodus rotundus* and *Pteronotus fulvus* (Phyllostomidae and Mormoopidae) and the EF1 genotype in *Glossophaga soricina* and *Saccopteryx bilineata* (Phyllostomidae and Emballonuridae). For the latter, both bat species co‐roost in the LAR, which suggests an ecological context for pathogen exposure over current timescales. However, other genetic markers (e.g., *rpoB*, *rpoC*, *gyrB*) would be necessary to infer contemporary cross‐species transmission (Kämpfer & Glaeser, 2012; Volokhov et al., 2012), as analysis of the 16S rRNA gene alone is insufficient for haemoplasma species identification (Volokhov et al., 2012). If haemoplasmas are more likely to specialize rather than expand their range into new and unrelated species, genotype sharing between unrelated bats could represent more transient spillovers (Bonneaud et al., 2019). As specialized pathogens could be more transmissible than generalists (Garamszegi, 2006), species with high infection prevalence of specialist genotypes could be prioritized for bacterial surveillance.

In conclusion, our analysis of a diverse community of bats and their pathogen genotypes identifies several key ecological and evolutionary factors structuring bacterial infection within and between species and provides a starting point for contrasts with such patterns for viruses. Similar to bat viruses, we found moderate phylogenetic signal in haemoplasma prevalence. However, these phylogenetic patterns in prevalence were decoupled from those describing bat species centrality in sharing haemoplasmas, such that genotype sharing was generally restricted by bat phylogeny. These findings imply codivergence of bats and their bacterial pathogens alongside rare and phylogenetically constrained host shifts. Future work more broadly characterizing the ecological and evolutionary determinants of bacterial infections in diverse host communities will improve our understanding of cross‐species transmission beyond viruses and contribute to efforts to understand the epidemiological consequences of bacterial pathogens.

## AUTHOR CONTRIBUTIONS

D.J.B., K.A.S. and A.M.B. collected samples; N.B.S. and M.B.F. coordinated fieldwork; R.K.P., S.A. and D.G.S. supported laboratory analyses; D.V.V. and V.E.C. conducted molecular and phylogenetic analyses; and A.D.W. and D.G.S. assisted with statistical analyses. D.J.B. analysed data, produced figures and wrote the manuscript. All authors provided critical review of the manuscript.

## CONFLICT OF INTEREST

We have no competing interests.

## Supporting information

Supplementary Material

## Data Availability

Individual‐level data are available in Dryad (https://doi.org/10.5061/dryad.j6q573n8r) (Becker et al., 2019). Bat species trait data are available in Table S2. Haemoplasma sequences are available in GenBank (MH245119–MH245194 and MK353807–MK353892).

## References

[mec15422-bib-0001] Albery, G. F. , Eskew, E. A. , Ross, N. , & Olival, K. J. (2019). Predicting the Global Mammalian Viral Sharing Network Using Phylogeography, BioRxiv, 732255. 10.1038/s41467-020-16153-4 PMC7210981 32385239

[mec15422-bib-0002] Alcorn, K. , Gerrard, J. , Cochrane, T. , Graham, R. , Jennison, A. , Irwin, P. J. , & Barbosa, A. D. (2020). First report of Candidatus Mycoplasma haemohominis infection in Australia causing persistent fever in an animal carer. Clinical Infectious Diseases. 10.1093/cid/ciaa089 32006025

[mec15422-bib-0003] Bai, Y. , Kosoy, M. , Recuenco, S. , Alvarez, D. , Moran, D. , Turmelle, A. , … Rupprecht, C. (2011). *Bartonella* spp. in bats, Guatemala. Emerging Infectious Diseases, 17(7), 1269–1272. 10.3201/eid1707.101867 21762584 PMC3381397

[mec15422-bib-0004] Baillie, J. , Hilton‐Taylor, C. , & Stuart, S. N. (2004). IUCN red list of threatened species: a global species assessment. Gland, Switzerland and Cambridge, UK:IUCN.

[mec15422-bib-0005] Balbuena, J. A. , Míguez‐Lozano, R. , & Blasco‐Costa, I. (2013). PACo: A novel Procrustes application to cophylogenetic analysis. PLoS ONE, 8(4), e61048. 10.1371/journal.pone.0061048 23580325 PMC3620278

[mec15422-bib-0006] Becker, D. J. , Bergner, L. M. , Bentz, A. B. , Orton, R. J. , Altizer, S. , & Streicker, D. G. (2018). Genetic diversity, infection prevalence, and possible transmission routes of *Bartonella* spp. in vampire bats. PLOS Neglected Tropical Diseases, 12(9), e0006786. 10.1371/journal.pntd.0006786 30260954 PMC6159870

[mec15422-bib-0007] Becker, D. J. , Crowley, D. E. , Washburne, A. D. , & Plowright, R. K. (2019). Temporal and spatial limitations in global surveillance for bat filoviruses and henipaviruses. Biology Letters, 15(12), 20190423. 10.1098/rsbl.2019.0423 31822244 PMC6936026

[mec15422-bib-0008] Becker, D. J. , Speer, K. A. , Brown, A. M. , Fenton, M. B. , Washburne, A. D. , Altizer, S. , … Volokhov, D. V. (2019). Ecological and evolutionary drivers of hemoplasma infection and genotype sharing in a Neotropical bat community. Dataset. 10.5061/dryad.j6q573n8r PMC8299350 32243630

[mec15422-bib-0009] Bell, D. C. , Atkinson, J. S. , & Carlson, J. W. (1999). Centrality measures for disease transmission networks. Social Networks, 21(1), 1–21. 10.1016/S0378-8733(98)00010-0

[mec15422-bib-0010] Blyton, M. D. J. , Banks, S. C. , Peakall, R. , Lindenmayer, D. B. , & Gordon, D. M. (2014). Not all types of host contacts are equal when it comes to *E. coli* transmission. Ecology Letters, 17(8), 970–978. 10.1111/ele.12300 24861219

[mec15422-bib-0011] Bonato, L. , Figueiredo, M. A. P. , Gonçalves, L. R. , Machado, R. Z. , & André, M. R. (2015). Occurrence and molecular characterization of *Bartonella* spp. and hemoplasmas in neotropical primates from Brazilian Amazon. Comparative Immunology, Microbiology and Infectious Diseases, 42, 15–20. 10.1016/j.cimid.2015.09.001 26577193

[mec15422-bib-0012] Bonneaud, C. , Weinert, L. , & Kuijper, B. (2019). Understanding the emergence of bacterial pathogens in novel hosts. Philosophical Transactions of the Royal Society B: Biological Sciences, 374(1782), 20180328. 10.1098/rstb.2018.0328 PMC6711297 31401968

[mec15422-bib-0013] Botero‐Castro, F. , Tilak, M. , Justy, F. , Catzeflis, F. , Delsuc, F. , & Douzery, E. J. P. (2013). Next‐generation sequencing and phylogenetic signal of complete mitochondrial genomes for resolving the evolutionary history of leaf‐nosed bats (Phyllostomidae). Molecular Phylogenetics and Evolution, 69(3), 728–739. 10.1016/j.ympev.2013.07.003 23850499

[mec15422-bib-0014] Bürkner, P.‐C. (2017). brms: An R package for Bayesian multilevel models using Stan. Journal of Statistical Software, 80(1), 1–28.

[mec15422-bib-0015] Burnham, K. P. , & Anderson, D. R. (2002). Model selection and multimodel inference: A practical information‐theoretic approach. New York, NY:Springer Science & Business Media.

[mec15422-bib-0016] Butts, C. T. (2008). Social network analysis with sna. Journal of Statistical Software, 24(6), 1–51. 18618019 10.18637/jss.v024.i01 PMC2447931

[mec15422-bib-0017] Chamberlain, S. A. , & Szöcs, E. (2013). Taxize: Taxonomic search and retrieval in R. F1000Research, 2, 191. 10.12688/f1000research.2-191.v1 24555091 PMC3901538

[mec15422-bib-0018] Citti, C. , & Blanchard, A. (2013). Mycoplasmas and their host: Emerging and re‐emerging minimal pathogens. Trends in Microbiology, 21(4), 196–203. 10.1016/j.tim.2013.01.003 23419218

[mec15422-bib-0019] Clare, E. L. , Lim, B. K. , Engstrom, M. D. , Eger, J. L. , & Hebert, P. D. (2007). DNA barcoding of Neotropical bats: Species identification and discovery within Guyana. Molecular Ecology Notes, 7(2), 184–190. 10.1111/j.1471-8286.2006.01657.x

[mec15422-bib-0020] Cohen, C. , Shemesh, M. , Garrido, M. , Messika, I. , Einav, M. , Khokhlova, I. , … Hawlena, H. (2018). Haemoplasmas in wild rodents: Routes of transmission and infection dynamics. Molecular Ecology, 27(18), 3714–3726. 10.1111/mec.14826 30074652

[mec15422-bib-0021] Csardi, G. , & Nepusz, T. (2006). The igraph software package for complex network research. InterJournal, Complex Systems, 1695(5), 1–9.

[mec15422-bib-0022] Dallas, T. A. , Han, B. A. , Nunn, C. L. , Park, A. W. , Stephens, P. R. , & Drake, J. M. (2019). Host traits associated with species roles in parasite sharing networks. Oikos, 128(1), 23–32. 10.1111/oik.05602

[mec15422-bib-0023] De Vienne, D. M. , Refrégier, G. , López‐Villavicencio, M. , Tellier, A. , Hood, M. E. , & Giraud, T. (2013). Cospeciation vs host‐shift speciation: Methods for testing, evidence from natural associations and relation to coevolution. New Phytologist, 198(2), 347–385. 10.1111/nph.12150 23437795

[mec15422-bib-0024] Di Cataldo, S. , Kamani, J. , Cevidanes, A. , Msheliza, E. G. , & Millán, J. (2020). Hemotropic mycoplasmas in bats captured near human settlements in Nigeria. Comparative Immunology, Microbiology and Infectious Diseases, 70, 101448. 10.1016/j.cimid.2020.101448 32109761

[mec15422-bib-0025] Downs, C. J. , Schoenle, L. A. , Han, B. A. , Harrison, J. F. , & Martin, L. B. (2019). Scaling of host competence. Trends in Parasitology, 35, 182–192. 10.1016/j.pt.2018.12.002 30709569

[mec15422-bib-0026] Edgar, R. C. , Haas, B. J. , Clemente, J. C. , Quince, C. , & Knight, R. (2011). UCHIME improves sensitivity and speed of chimera detection. Bioinformatics, 27(16), 2194–2200. 10.1093/bioinformatics/btr381 21700674 PMC3150044

[mec15422-bib-0027] Evans, N. J. , Bown, K. , Timofte, D. , Simpson, V. R. , & Birtles, R. J. (2009). Fatal borreliosis in bat caused by relapsing fever spirochete, Unoted Kingdom. Emerging Infectious Diseases, 15(8), 1331–1333. 10.3201/eid1508.090475 19751613 PMC2815988

[mec15422-bib-0028] Fenton, A. , Streicker, D. G. , Petchey, O. L. , & Pedersen, A. B. (2015). Are all hosts created equal? Partitioning host species contributions to parasite persistence in multihost communities. The American Naturalist, 186(5), 610–622. 10.1086/683173 PMC6542667 26655774

[mec15422-bib-0029] Fenton, M. B. , Bernard, E. , Bouchard, S. , Hollis, L. , Johnston, D. S. , Lausen, C. L. , … Zigouris, J. (2001). The bat fauna of Lamanai, Belize: Roosts and trophic roles. Journal of Tropical Ecology, 17(4), 511–524. 10.1017/S0266467401001389

[mec15422-bib-0030] Fountain‐Jones, N. M. , Pearse, W. D. , Escobar, L. E. , Alba‐Casals, A. , Carver, S. , Davies, T. J. , … Craft, M. E. (2018). Towards an eco‐phylogenetic framework for infectious disease ecology. Biological Reviews, 93(2), 950–970. 10.1111/brv.12380 29114986

[mec15422-bib-0031] Frank, H. K. , Mendenhall, C. D. , Judson, S. D. , Daily, G. C. , & Hadly, E. A. (2016). Anthropogenic impacts on Costa Rican bat parasitism are sex specific. Ecology and Evolution, 6(14), 4898–4909. 10.1002/ece3.2245 27547321 PMC4979715

[mec15422-bib-0032] Garamszegi, L. Z. (2006). The evolution of virulence and host specialization in malaria parasites of primates. Ecology Letters, 9(8), 933–940. 10.1111/j.1461-0248.2006.00936.x 16913936

[mec15422-bib-0033] Garamszegi, L. Z. (2014). Modern phylogenetic comparative methods and their application in evolutionary biology: Concepts and practice. Berlin, Germany:Springer.

[mec15422-bib-0034] Gelman, A. , Goodrich, B. , Gabry, J. , & Vehtari, A. (2019). R‐squared for Bayesian regression models. The American Statistician, 73(3), 307–309. 10.1080/00031305.2018.1549100

[mec15422-bib-0035] Geoghegan, J. L. , Duchêne, S. , & Holmes, E. C. (2017). Comparative analysis estimates the relative frequencies of co‐divergence and cross‐species transmission within viral families. PLoS Path, 13(2), e1006215. 10.1371/journal.ppat.1006215 PMC5319820 28178344

[mec15422-bib-0036] Gómez, J. M. , Nunn, C. L. , & Verdú, M. (2013). Centrality in primate–parasite networks reveals the potential for the transmission of emerging infectious diseases to humans. Proceedings of the National Academy of Sciences of the United States of America, 110(19), 7738–7741. 10.1073/pnas.1220716110 23610389 PMC3651426

[mec15422-bib-0037] Gonçalves, L. R. , Roque, A. L. R. , Matos, C. A. , Fernandes, S. D. J. , Olmos, I. D. F. , Machado, R. Z. , & André, M. R. (2015). Diversity and molecular characterization of novel hemoplasmas infecting wild rodents from different Brazilian biomes. Comparative Immunology, Microbiology and Infectious Diseases, 43, 50–56. 10.1016/j.cimid.2015.10.006 26616660

[mec15422-bib-0038] González‐Salazar, C. , Martínez‐Meyer, E. , & López‐Santiago, G. (2014). A hierarchical classification of trophic guilds for North American birds and mammals. Revista Mexicana De Biodiversidad, 85(3), 931–941. 10.7550/rmb.38023

[mec15422-bib-0039] Goto, K. , Yasuda, M. , Hayashimoto, N. , & Ebukuro, S. (2010). Morphological and sequence analysis of *Mycoplasma* sp. isolated from the oral cavity of a house musk shrew (*Suncus murinus*). The Journal of Veterinary Medical Science, 72(1), 109–111. 19915329 10.1292/jvms.09-0307

[mec15422-bib-0040] Graham, C. H. , Storch, D. , & Machac, A. (2018). Phylogenetic scale in ecology and evolution. Global Ecology and Biogeography, 27(2), 175–187. 10.1111/geb.12686

[mec15422-bib-0041] Gunnell, G. F. , & Simmons, N. B. (2012). Evolutionary history of bats: Fossils, molecules and morphology. Cambridge: Cambridge University Press.

[mec15422-bib-0042] Guy, C. , Thiagavel, J. , Mideo, N. , & Ratcliffe, J. M. (2019). Phylogeny matters: Revisiting ‘a comparison of bats and rodents as reservoirs of zoonotic viruses’. Royal Society Open Science, 6(2), 181182. 10.1098/rsos.181182 30891262 PMC6408376

[mec15422-bib-0043] Han, B. A. , Kramer, A. M. , & Drake, J. M. (2016). Global patterns of zoonotic disease in mammals. Trends in Parasitology, 32(7), 565–577. 10.1016/j.pt.2016.04.007 27316904 PMC4921293

[mec15422-bib-0044] Han, B. A. , Schmidt, J. P. , Alexander, L. W. , Bowden, S. E. , Hayman, D. T. , & Drake, J. M. (2016). Undiscovered bat hosts of filoviruses. PLoS Neglected Tropical Diseases, 10(7), e0004815. 10.1371/journal.pntd.0004815 27414412 PMC4945033

[mec15422-bib-0045] Hattori, N. , Kuroda, M. , Katano, H. , Takuma, T. , Ito, T. , Arai, N. , … Niki, Y. (2020). Candidatus *Mycoplasma haemohominis* in human, Japan. Emerging Infectious Diseases, 26(1), 11–19. 10.3201/eid2601.190983 31855136 PMC6924906

[mec15422-bib-0046] Herrera, J. P. , Duncan, N. , Clare, E. , Fenton, M. B. , & Simmons, N. (2018). Disassembly of fragmented bat communities in Orange Walk District, Belize. Acta Chiropterologica, 20(1), 147–159. 10.3161/15081109ACC2018.20.1.011

[mec15422-bib-0047] Hijmans, R. J. , Williams, E. , Vennes, C. , & Hijmans, M. R. J. (2019). Package ‘geosphere’. Spherical Trigonometryv,1–5.

[mec15422-bib-0048] Hochachka, W. M. , & Dhondt, A. A. (2000). Density‐dependent decline of host abundance resulting from a new infectious disease. Proceedings of the National Academy of Sciences of the United States of America, 97(10), 5303–5306. 10.1073/pnas.080551197 10792031 PMC25823

[mec15422-bib-0049] Hornok, S. , Szőke, K. , Meli, M. L. , Sándor, A. D. , Görföl, T. , Estók, P. , … Hofmann‐Lehmann, R. (2019). Molecular detection of vector‐borne bacteria in bat ticks (Acari: Ixodidae, Argasidae) from eight countries of the old and new worlds. Parasites & Vectors, 12(1), 50. 10.1186/s13071-019-3303-4 30670048 PMC6343265

[mec15422-bib-0050] Huang, S. , Bininda‐Emonds, O. R. , Stephens, P. R. , Gittleman, J. L. , & Altizer, S. (2014). Phylogenetically related and ecologically similar carnivores harbour similar parasite assemblages. Journal of Animal Ecology, 83(3), 671–680. 10.1111/1365-2656.12160 24289314

[mec15422-bib-0051] Huang, S. , Drake, J. M. , Gittleman, J. L. , & Altizer, S. (2015). Parasite diversity declines with host evolutionary distinctiveness: A global analysis of carnivores. Evolution, 69(3), 621–630. 10.1111/evo.12611 25639279

[mec15422-bib-0052] Hutchinson, M. C. , Cagua, E. F. , Balbuena, J. A. , Stouffer, D. B. , & Poisot, T. (2017). paco: Implementing Procrustean approach to cophylogeny in R. Methods in Ecology and Evolution, 8(8), 932–940. 10.1111/2041-210X.12736

[mec15422-bib-0053] Ikeda, P. , Seki, M. C. , Carrasco, A. O. T. , Rudiak, L. V. , Miranda, J. M. D. , Gonçalves, S. M. M. … André, M. R. (2017). Evidence and molecular characterization of *Bartonella* spp. and hemoplasmas in neotropical bats in Brazil. Epidemiology & Infection, 145(10), 2038–2052. 28502279 10.1017/S0950268817000966 PMC9203434

[mec15422-bib-0054] Johnson, P. T. J. , Rohr, J. R. , Hoverman, J. T. , Kellermanns, E. , Bowerman, J. , & Lunde, K. B. (2012). Living fast and dying of infection: Host life history drives interspecific variation in infection and disease risk. Ecology Letters, 15(3), 235–242. 10.1111/j.1461-0248.2011.01730.x 22221837

[mec15422-bib-0055] Jombart, T. , Kendall, M. , Almagro‐Garcia, J. , & Colijn, C. (2017). treespace: Statistical exploration of landscapes of phylogenetic trees. Molecular Ecology Resources, 17(6), 1385–1392. 10.1111/1755-0998.12676 28374552 PMC5724650

[mec15422-bib-0056] Jones, P. L. , Hämsch, F. , Page, R. A. , Kalko, E. K. , & Omara, M. T. (2017). Foraging and roosting behaviour of the fringe‐lipped bat, *Trachops cirrhosus*, on Barro Colorado Island. Panamá. Acta Chiropterologica, 19(2), 337–346.

[mec15422-bib-0057] Kämpfer, P. , & Glaeser, S. P. (2012). Prokaryotic taxonomy in the sequencing era–the polyphasic approach revisited. Environmental Microbiology, 14(2), 291–317. 10.1111/j.1462-2920.2011.02615.x 22040009

[mec15422-bib-0058] Kellner, A. , Carver, S. , Scorza, V. , McKee, C. D. , Lappin, M. , Crooks, K. R. , … Antolin, M. F. (2018). Transmission pathways and spillover of an erythrocytic bacterial pathogen from domestic cats to wild felids. Ecology and Evolution, 8(19), 9779–9792. 10.1002/ece3.4451 30386574 PMC6202716

[mec15422-bib-0059] Kelly, C. D. , Stoehr, A. M. , Nunn, C. , Smyth, K. N. , & Prokop, Z. M. (2018). Sexual dimorphism in immunity across animals: A meta‐analysis. Ecology Letters, 21(12), 1885–1894. 10.1111/ele.13164 30288910

[mec15422-bib-0060] Kembel, S. W. , Cowan, P. D. , Helmus, M. R. , Cornwell, W. K. , Morlon, H. , Ackerly, D. D. , … Webb, C. O. (2010). Picante: R tools for integrating phylogenies and ecology. Bioinformatics, 26(11), 1463–1464. 10.1093/bioinformatics/btq166 20395285

[mec15422-bib-0061] Kumar, S. , Stecher, G. , Li, M. , Knyaz, C. , & Tamura, K. (2018). MEGA X: Molecular evolutionary genetics analysis across computing platforms. Molecular Biology and Evolution, 35(6), 1547–1549. 10.1093/molbev/msy096 29722887 PMC5967553

[mec15422-bib-0062] Loayza, A. P. , & Loiselle, B. A. (2008). Preliminary information on the home range and movement patterns of *Sturnira lilium* (Phyllostomidae) in a naturally fragmented landscape in Bolivia. Biotropica, 40(5), 630–635.

[mec15422-bib-0063] Longdon, B. , Brockhurst, M. A. , Russell, C. A. , Welch, J. J. , & Jiggins, F. M. (2014). The evolution and genetics of virus host shifts. PLoS Path, 10(11), e1004395. 10.1371/journal.ppat.1004395 PMC4223060 25375777

[mec15422-bib-0064] Luis, A. D. , O'Shea, T. J. , Hayman, D. T. S. , Wood, J. L. N. , Cunningham, A. A. , Gilbert, A. T. , … Webb, C. T. (2015). Network analysis of host–virus communities in bats and rodents reveals determinants of cross‐species transmission. Ecology Letters, 18(11), 1153–1162. 10.1111/ele.12491 26299267 PMC5014217

[mec15422-bib-0065] Maggi, R. G. , Compton, S. M. , Trull, C. L. , Mascarelli, P. E. , Mozayeni, B. R. , & Breitschwerdt, E. B. (2013). Infection with hemotropic *Mycoplasma* species in patients with or without extensive arthropod or animal contact. Journal of Clinical Microbiology, 51(10), 3237–3241. 10.1128/JCM.01125-13 23863574 PMC3811635

[mec15422-bib-0066] Mascarelli, P. E. , Keel, M. K. , Yabsley, M. , Last, L. A. , Breitschwerdt, E. B. , & Maggi, R. G. (2014). Hemotropic mycoplasmas in little brown bats (*Myotis lucifugu*s). Parasit Vectors, 7, 117. 10.1186/1756-3305-7-117 24655520 PMC3994326

[mec15422-bib-0067] McCallum, H. , Barlow, N. , & Hone, J. (2001). How should pathogen transmission be modelled? Trends in Ecology & Evolution, 16(6), 295–300. 10.1016/S0169-5347(01)02144-9 11369107

[mec15422-bib-0068] McKee, C. D. , Krawczyk, A. I. , Sándor, A. , Görföl, T. , Földvári, M. , & Földvári, G. (2019). Host phylogeny, geographic overlap, and roost sharing shape parasite communities in European bats. Frontiers in Ecology and Evolution, 7, 69.

[mec15422-bib-0069] Messick, J. B. (2004). Hemotrophic mycoplasmas (hemoplasmas): A review and new insights into pathogenic potential. Veterinary Clinical Pathology, 33(1), 2–13. 10.1111/j.1939-165X.2004.tb00342.x 15048620

[mec15422-bib-0070] Michonneau, F. , Brown, J. W. , & Winter, D. J. (2016). rotl : An R package to interact with the Open Tree of Life data. Methods in Ecology and Evolution, 7(12), 1476–1481. 10.1111/2041-210X.12593

[mec15422-bib-0071] Millán, J. , Cevidanes, A. , Sacristán, I. , Alvarado, M. , Sepúlveda, G. , Ramos‐Mella, C. A. , & Lisón, F. (2019). Detection and characterization of hemotropic mycoplasmas in bats in Chile. Journal of Wildlife Diseases, 55, 977. 10.7589/2018-12-290 31009305

[mec15422-bib-0072] Millán, J. , López‐Roig, M. , Delicado, V. , Serra‐Cobo, J. , & Esperón, F. (2015). Widespread infection with hemotropic mycoplasmas in bats in Spain, including a hemoplasma closely related to “Candidatus Mycoplasma hemohominis”. Comparative Immunology, Microbiology and Infectious Diseases, 39, 9–12. 10.1016/j.cimid.2015.01.002 25655409

[mec15422-bib-0073] Monteiro, L. R. , & Nogueira, M. R. (2011). Evolutionary patterns and processes in the radiation of phyllostomid bats. BMC Evolutionary Biology, 11, 137. 10.1186/1471-2148-11-137 21605452 PMC3130678

[mec15422-bib-0074] Mühldorfer, K. (2013). Bats and bacterial pathogens: A review. Zoonoses and Public Health, 60(1), 93–103. 10.1111/j.1863-2378.2012.01536.x 22862791

[mec15422-bib-0075] Museux, K. , Boretti, F. S. , Willi, B. , Riond, B. , Hoelzle, K. , Hoelzle, L. E. , … Hofmann‐Lehmann, R. (2009). In vivo transmission studies of “Candidatus *Mycoplasma turicensis*” in the domestic cat. Veterinary Research, 40(5), 45. 10.1051/vetres/2009028 19505421 PMC2701178

[mec15422-bib-0076] Myhrvold, N. P. , Baldridge, E. , Chan, B. , Sivam, D. , Freeman, D. L. , & Ernest, S. K. M. (2015). An amniote life‐history database to perform comparative analyses with birds, mammals, and reptiles. Ecology, 96(11), 3109–3109. 10.1890/15-0846R.1

[mec15422-bib-0077] Olival, K. J. , Hosseini, P. R. , Zambrana‐Torrelio, C. , Ross, N. , Bogich, T. L. , & Daszak, P. (2017). Host and viral traits predict zoonotic spillover from mammals. Nature, 546(7660), 646–650. 10.1038/nature22975 28636590 PMC5570460

[mec15422-bib-0078] Paradis, E. , Claude, J. , & Strimmer, K. (2004). APE: Analyses of phylogenetics and evolution in R language. Bioinformatics, 20(2), 289–290. 10.1093/bioinformatics/btg412 14734327

[mec15422-bib-0079] Pedersen, A. B. , Altizer, S. , Poss, M. , Cunningham, A. A. , & Nunn, C. L. (2005). Patterns of host specificity and transmission among parasites of wild primates. International Journal for Parasitology, 35(6), 647–657. 10.1016/j.ijpara.2005.01.005 15862578

[mec15422-bib-0080] Pitcher, D. G. , & Nicholas, R. A. J. (2005). Mycoplasma host specificity: Fact or fiction? The Veterinary Journal, 170(3), 300–306. 10.1016/j.tvjl.2004.08.011 16266844

[mec15422-bib-0081] Plowright, R. K. , Parrish, C. R. , McCallum, H. , Hudson, P. J. , Ko, A. I. , Graham, A. L. , & Lloyd‐Smith, J. O. (2017). Pathways to zoonotic spillover. Nature Reviews Microbiology, 15(8), 502–510. 10.1038/nrmicro.2017.45 28555073 PMC5791534

[mec15422-bib-0082] Reid, F. (1997). A field guide to the mammals of central America and Southeast Mexico. New York, NY: Oxford University Press.

[mec15422-bib-0083] Santana, S. E. , Dial, T. O. , Eiting, T. P. , & Alfaro, M. E. (2011). Roosting ecology and the evolution of pelage markings in bats. PLoS ONE, 6(10), e25845. 10.1371/journal.pone.0025845 21991371 PMC3185059

[mec15422-bib-0084] Sikes, R. S. & the Animal Care and Use Committee of the American Society of Mammalogists (2016). 2016 Guidelines of the American Society of Mammalogists for the use of wild mammals in research and education. Journal of Mammalogy, 97(3), 663–688. 10.1093/jmammal/gyw078 29692469 PMC5909806

[mec15422-bib-0085] Soto, F. , Walker, R. , Sepulveda, M. , Bittencourt, P. , Acosta‐Jamett, G. , & Müller, A. (2017). Occurrence of canine hemotropic mycoplasmas in domestic dogs from urban and rural areas of the Valdivia Province, southern Chile. Comparative Immunology, Microbiology and Infectious Diseases, 50, 70–77. 10.1016/j.cimid.2016.11.013 28131382

[mec15422-bib-0086] Streicker, D. G. , Recuenco, S. , Valderrama, W. , Gomez Benavides, J. , Vargas, I. , Pacheco, V. , … Altizer, S. (2012). Ecological and anthropogenic drivers of rabies exposure in vampire bats: Implications for transmission and control. Proceedings of the Royal Society B, 279(1742), 3384–3392. 10.1098/rspb.2012.0538 22696521 PMC3396893

[mec15422-bib-0087] Streicker, D. G. , Turmelle, A. S. , Vonhof, M. J. , Kuzmin, I. V. , McCracken, G. F. , & Rupprecht, C. E. (2010). Host phylogeny constrains cross‐species emergence and establishment of rabies virus in bats. Science, 329(5992), 676–679. 20689015 10.1126/science.1188836

[mec15422-bib-0088] Ter Hofstede, H. M. , Fenton, M. B. , & Whitaker, J. O. (2004). Host and host‐site specificity of bat flies (Diptera: Streblidae and Nycteribiidae) on Neotropical bats (Chiroptera). Canadian Journal of Zoology, 82(4), 616–626. 10.1139/z04-030

[mec15422-bib-0089] VanderWaal, K. L. , Atwill, E. R. , Isbell, L. A. , & McCowan, B. (2014). Quantifying microbe transmission networks for wild and domestic ungulates in Kenya. Biological Conservation, 169(Supplement C), 136–146. 10.1016/j.biocon.2013.11.008

[mec15422-bib-0090] VanderWaal, K. L. , & Ezenwa, V. O. (2016). Heterogeneity in pathogen transmission: Mechanisms and methodology. Functional Ecology, 30(10), 1606–1622. 10.1111/1365-2435.12645

[mec15422-bib-0091] Vehtari, A. , Gelman, A. , & Gabry, J. (2017). Practical Bayesian model evaluation using leave‐one‐out cross‐validation and WAIC. Statistics and Computing, 27(5), 1413–1432. 10.1007/s11222-016-9696-4

[mec15422-bib-0092] Viechtbauer, W. (2010). Conducting meta‐analyses in R with the metafor package. Journal of Statistical Software, 36(3), 1–48.

[mec15422-bib-0093] Vieira, R. F. C. , Molento, M. B. , dos Santos, L. C. , Moraes, W. , Cubas, Z. S. , Santos, A. P. , … Messick, J. B. (2009). Detection of a novel hemoplasma based on 16S rRNA gene DNA in captive and free‐ranging capybaras (*Hydrochaeris hydrochaeris*). Veterinary Microbiology, 139(3–4), 410–413. 10.1016/j.vetmic.2009.06.018 19592180

[mec15422-bib-0094] Voigt, C. C. , von Helversen, O. , Michener, R. , & Kunz, T. H. (2001). The economics of harem maintenance in the sac‐winged bat, *Saccopteryx bilineata* (Emballonuridae). Behavioral Ecology and Sociobiology, 50(1), 31–36. 10.1007/s002650100337

[mec15422-bib-0095] Volokhov, D. V. , Becker, D. J. , Bergner, L. M. , Camus, M. S. , Orton, R. J. , Chizhikov, V. E. , … Streicker, D. G. (2017). Novel hemotropic mycoplasmas are widespread and genetically diverse in vampire bats. Epidemiology & Infection, 145(15), 3154–3167. 10.1017/S095026881700231X 29061202 PMC6538534

[mec15422-bib-0096] Volokhov, D. V. , Hwang, J. , Chizhikov, V. E. , Danaceau, H. , & Gottdenker, N. L. (2017). Prevalence, genotype richness, and coinfection patterns of hemotropic mycoplasmas in raccoons (*Procyon lotor*) on environmentally protected and urbanized barrier islands. Applied and Environmental Microbiology, 83(9), e00211–17. 10.1128/AEM.00211-17 28258139 PMC5394313

[mec15422-bib-0097] Volokhov, D. V. , Kong, H. , George, J. , Anderson, C. , & Chizhikov, V. E. (2008). Biological enrichment of Mycoplasma agents by cocultivation with permissive cell cultures. Applied and Environmental Microbiology, 74(17), 5383–5391. 10.1128/AEM.00720-08 18606798 PMC2546620

[mec15422-bib-0098] Volokhov, D. V. , Norris, T. , Rios, C. , Davidson, M. K. , Messick, J. B. , Gulland, F. M. , & Chizhikov, V. E. (2011). Novel hemotrophic mycoplasma identified in naturally infected California sea lions (*Zalophus californianus*). Veterinary Microbiology, 149(1–2), 262–268. 10.1016/j.vetmic.2010.10.026 21111543

[mec15422-bib-0099] Volokhov, D. V. , Simonyan, V. , Davidson, M. K. , & Chizhikov, V. E. (2012). RNA polymerase beta subunit (rpoB) gene and the 16S–23S rRNA intergenic transcribed spacer region (ITS) as complementary molecular markers in addition to the 16S rRNA gene for phylogenetic analysis and identification of the species of the family Mycoplasmataceae. Molecular Phylogenetics and Evolution, 62(1), 515–528. 10.1016/j.ympev.2011.11.002 22115576

[mec15422-bib-0100] Walker Vergara, R. , Morera Galleguillos, F. , Gómez Jaramillo, M. , Pereira Almosny, N. R. , Arauna Martínez, P. , Grob Behne, P. , … Müller, A. (2016). Prevalence, risk factor analysis, and hematological findings of hemoplasma infection in domestic cats from Valdivia, Southern Chile. Comparative Immunology, Microbiology and Infectious Diseases, 46, 20–26. 10.1016/j.cimid.2016.03.004 27260806

[mec15422-bib-0101] Washburne, A. D. , Silverman, J. D. , Morton, J. T. , Becker, D. J. , Crowley, D. , Mukherjee, S. , … Plowright, R. K. (2019). Phylofactorization: A graph partitioning algorithm to identify phylogenetic scales of ecological data. Ecological Monographs, 89, e01353. 10.1002/ecm.1353

[mec15422-bib-0102] Webber, Q. M. R. , Fletcher, Q. E. , & Willis, C. K. R. (2017). Viral richness is positively related to group size, but not mating system, in bats. EcoHealth, 14(4), 652–661. 10.1007/s10393-017-1276-3 29030788

[mec15422-bib-0103] Willi, B. , Boretti, F. S. , Meli, M. L. , Bernasconi, M. V. , Casati, S. , Hegglin, D. , … Hofmann‐Lehmann, R. (2007). Real‐time PCR investigation of potential vectors, reservoirs, and shedding patterns of feline hemotropic mycoplasmas. Applied and Environmental Microbiology, 73(12), 3798–3802. 10.1128/AEM.02977-06 17468284 PMC1932730

[mec15422-bib-0104] Willi, B. , Boretti, F. S. , Tasker, S. , Meli, M. L. , Wengi, N. , Reusch, C. E. , … Hofmann‐Lehmann, R. (2007). From *Haemobartonella* to hemoplasma: Molecular methods provide new insights. Veterinary Microbiology, 125(3–4), 197–209. 10.1016/j.vetmic.2007.06.027 17706380

[mec15422-bib-0105] Wilman, H. , Belmaker, J. , Simpson, J. , de la Rosa, C. , Rivadeneira, M. M. , & Jetz, W. (2014). EltonTraits 1.0: Species‐level foraging attributes of the world’s birds and mammals. Ecology, 95(7), 2027–2027. 10.1890/13-1917.1

[mec15422-bib-0106] Withenshaw, S. M. , Devevey, G. , Pedersen, A. B. , & Fenton, A. (2016). Multihost *Bartonella* parasites display covert host specificity even when transmitted by generalist vectors. Journal of Animal Ecology, 85(6), 1442–1452. 10.1111/1365-2656.12568 PMC5082552 27380876

[mec15422-bib-0107] Woolhouse, M. E. J. , & Gowtage‐Sequeria, S. (2005). Host range and emerging and reemerging pathogens. Emerging Infectious Diseases, 11(12), 1842–1847. 10.3201/eid1112.050997 16485468 PMC3367654

[mec15422-bib-0108] Woolhouse, M. E. , Taylor, L. H. , & Haydon, D. T. (2001). Population biology of multihost pathogens. Science, 292(5519), 1109–1112. 11352066 10.1126/science.1059026

[mec15422-bib-0109] Wright, E. S. , Yilmaz, L. S. , & Noguera, D. R. (2012). DECIPHER, a search‐based approach to chimera identification for 16S rRNA sequences. Applied and Environmental Microbiology, 78(3), 717–725. 10.1128/AEM.06516-11 22101057 PMC3264099

