# Supplementary material for: Ecological and evolutionary drivers of haemoplasma infection and bacterial genotype sharing in a Neotropical bat community
Source: Mol Ecol. 2020 Apr 21;29(8):1534–49. doi: 10.1111/mec.15422 (PMC8299350; doi:10.1111/mec.15422)

Supporting Information: Ecological and evolutionary drivers of hemoplasma infection and bacterial genotype sharing in a Neotropical bat community

Daniel J. Becker, Kelly A. Speer, Alexis M. Brown, M. Brock Fenton, Alex D. Washburne, Sonia Altizer, Daniel G. Streicker, Raina K. Plowright, Vladimir E. Chizhikov, Nancy B. Simmons, Dmitriy V. Volokhov

S1. Bat sampling

S2. Comparative data

S3. Infection status and prevalence

S4. Hemoplasma genotypes

S5. Ectoparasitism

S1. Bat sampling

Table S1. Sample size per bat species included in analyses of hemoplasma infection, stratified by year and site (LAR and KK).

	2017		2018	
**Species**	**LAR**	**KK**	**LAR**	**KK**
Artibeus intermedius	3	0	2	1
Artibeus jamaicensis	4	1	6	0
Artibeus lituratus	3	0	4	1
Bauerus dubiaquercus	4	0	2	0
Carollia perspicillata	3	2	2	1
Carollia sowelli	11	0	8	1
Chrotopterus auritus	0	3	1	0
Dermanura phaeotis	8	0	7	3
Dermanura watsoni	6	1	5	2
Desmodus rotundus	22	21	66	27
Eptesicus furinalis	8	1	10	0
Eumops nanus	1	0	0	0
Gardnerycteris keenani	0	0	2	0
Glossophaga soricina	11	0	7	0
Lasiurus ega	0	0	3	0
Lophostoma evotis	1	0	2	0
Micronycteris schmidtorum	1	0	0	0
Mimon cozumelae	1	0	0	5
Molossus nigricans	14	0	7	0
Mormoops megalophylla	0	0	1	1
Myotis elegans	0	0	2	0
Myotis pilosatibialis	1	0	3	0
Natalus mexicanus	0	0	1	0
Noctilio leporinus	0	0	1	0
Platyrrhinus helleri	0	0	1	1
Pteronotus fulvus	4	1	2	0
Pteronotus mesoamericanus	11	12	13	6
Rhogeesa aeneus	0	0	1	0
Rhynchonycteris naso	5	0	11	0
Saccopteryx bilineata	9	0	9	0
Sturnira parvidens	21	2	20	4
Trachops cirrhosus	0	3	4	1
Uroderma convexum	2	0	8	0

S2. Comparative data

Figure S1. Pairwise phylogenetic distance between the 33 bat species included in our dataset.

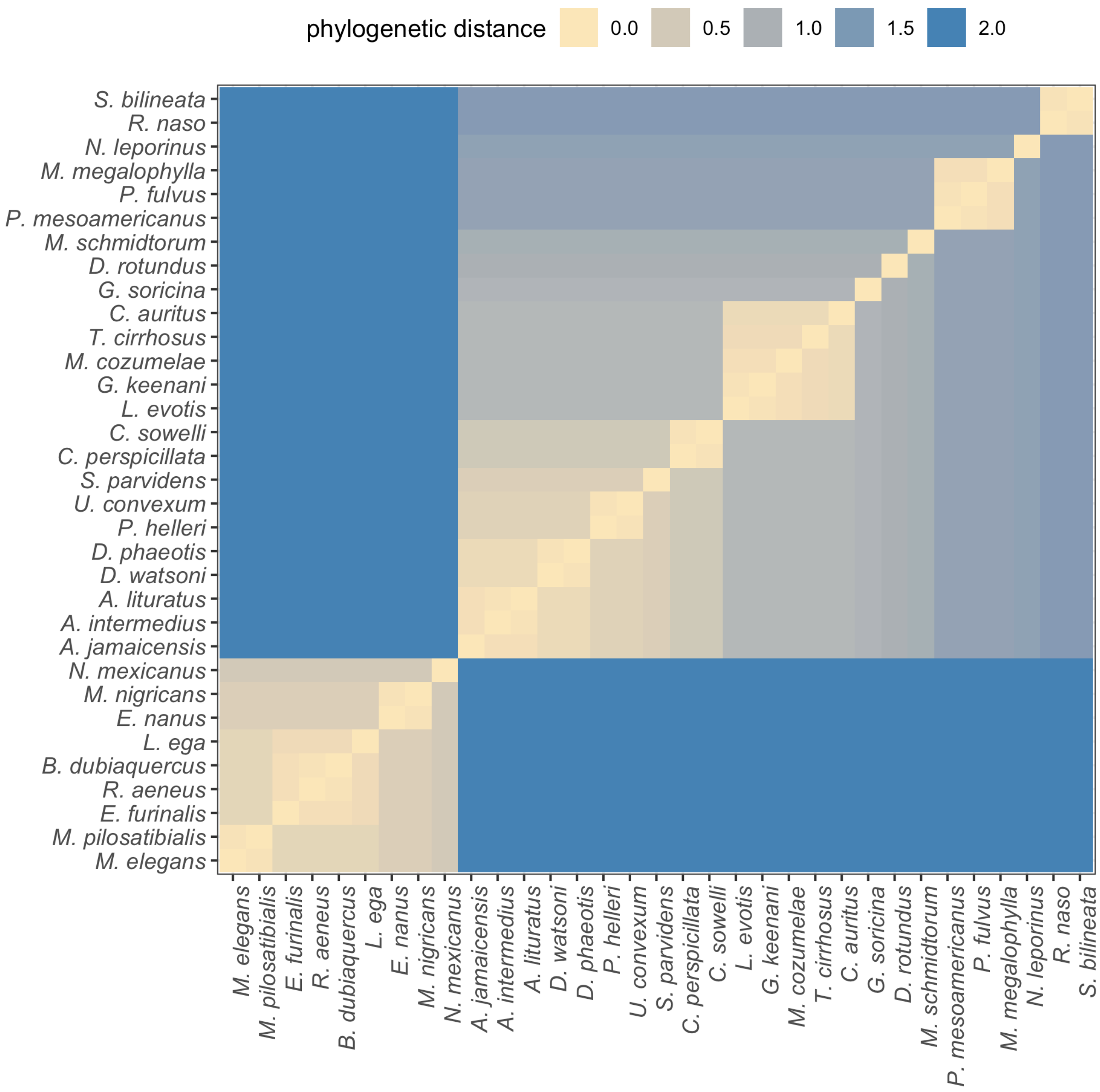

Figure S2. Map of geographic ranges for the 33 bat species sampled in Belize, stratified by whether hemoplasmas were detected. Transparent shapefiles from the International Union for Conservation of Nature are overlaid to highlight regions with dense distributional overlap.

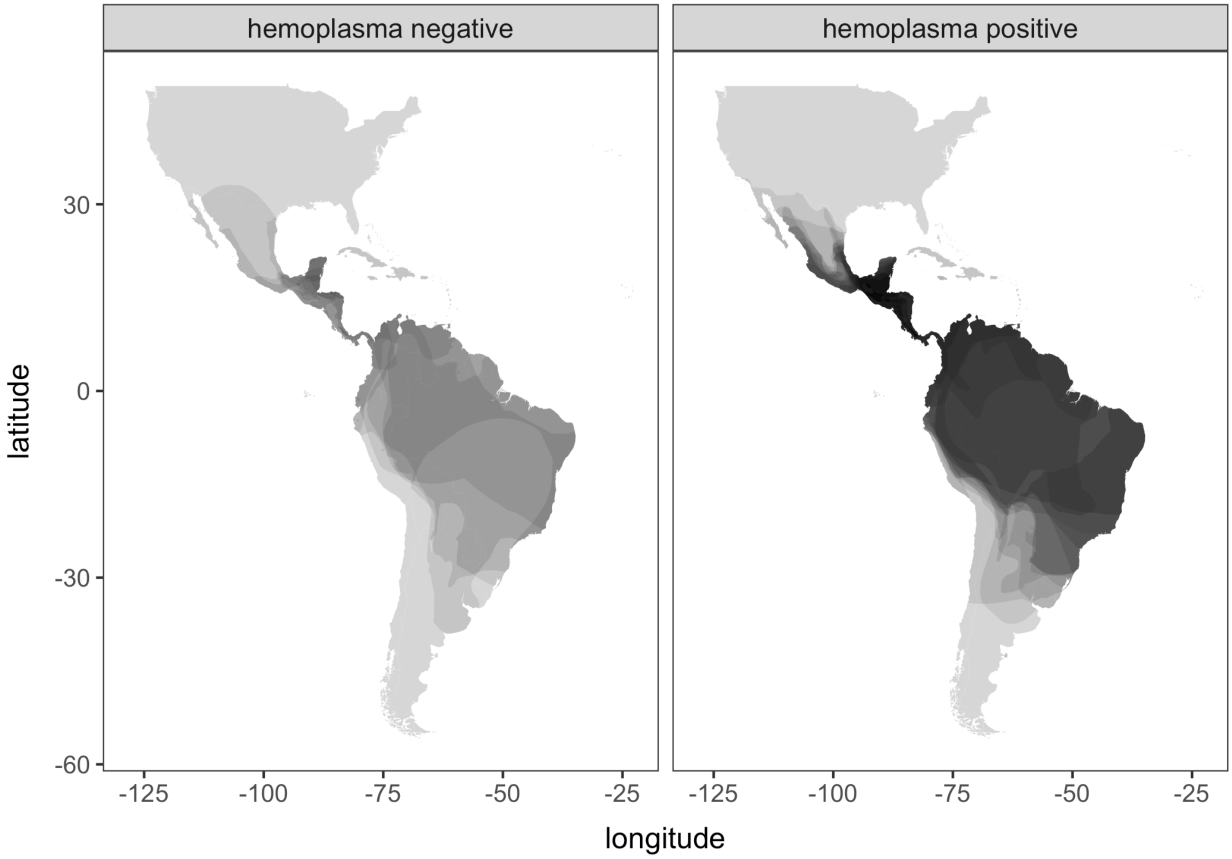

Table S2. Ecological and evolutionary trait data for the 33 Neotropical bat species included in phylogenetic comparative analyses.

Species		*N*	Mass	AF	Guild	% plant	Strata	AR	Roost	RF	Colony	KM^2	ED
Ai	*Artibeus intermedius*	6	60.5	1+	plant	90	arboreal	6.1	closed	1+	small	14235952	0.08
Aj	*Artibeus jamaicensis*	11	42.2	1+	plant	90	arboreal	6.4	closed	1+	small	1559896	0.12
Al	*Artibeus lituratus*	8	60.5	1+	plant	90	arboreal	6.1	closed	1+	small	14235952	0.08
Bd	*Bauerus dubiaquercus*	6	22.2	1+	insect	0	aerial	6.1	closed	1	small	265235	0.08
Cp	*Carollia perspicillata*	8	18	1+	plant	100	arboreal	6.1	closed	1+	large	13796364	0.20
Cs	*Carollia sowelli*	20	16.5	1+	plant	100	arboreal	5.5	closed	1+	large	790178	0.20
Ca	*Chrotopterus auritus*	4	77.7	1	carnivore	30	arboreal	5.5	closed	1+	small	13116793	0.30
Dp	*Dermanura phaeotis*	18	11.6	1+	plant	90	arboreal	6.3	open	1	small	3737367	0.11
Dw	*Dermanura watsoni*	14	11.2	1+	plant	90	arboreal	5.9	open	1	small	554677	0.11
Dr	*Desmodus rotundus*	139	31.2	1+	carnivore	0	ground/aquatic	6.7	closed	1+	large	17726518	0.56
Ef	*Eptesicus furinalis*	19	7.74	1+	insect	0	aerial	6.2	closed	1+	large	16292708	0.13
En	*Eumops nanus*	1	11.9	1	insect	0	aerial	8.8	closed	1	small	48804	0.23
Gk	*Gardnerycteris keenani*	2	13.8	1	insect	50	arboreal	8.3	NA	1	small	11136331	0.08
Gs	*Glossophaga soricina*	18	9.4	1+	plant	60	arboreal	6.4	closed	1+	medium	14858410	0.51
Lega	*Lasiurus ega*	3	13.2	1+	insect	0	aerial	7.9	closed	1+	small	13205183	0.19
Le	*Lophostoma evotis*	3	20.6	1	insect	10	arboreal	5.3	closed	1+	small	176593	0.08
Ms	*Micronycteris schmidtorum*	1	7.5	1	insect	20	arboreal	5.7	closed	1	small	6504802	0.61
Mc	*Mimon cozumelae*	6	18.6	1	insect	30	arboreal	8.3	closed	1+	small	803519	0.13
Mn	*Molossus nigricans*	21	25	1+	insect	0	aerial	11.1	closed	1	large	14800275	0.23
Mm	*Mormoops megalophylla*	2	15.8	1	insect	0	aerial	7.1	closed	1	large	3738686	0.38
Me	*Myotis elegans*	2	4.1	1	insect	0	aerial	6.4	closed	1	large	587280	0.16
Mp	*Myotis pilosatibialis*	4	5.3	1	insect	0	aerial	6.4	closed	1	large	2736294	0.16
Nm	*Natalus mexicanus*	1	5.5	1	insect	0	aerial	5.8	closed	1	large	2433276	0.63
Nl	*Noctilio leporinus*	1	58.3	1	carnivore	0	ground/aquatic	9	closed	1+	large	14625648	0.76
Ph	*Platyrrhinus helleri*	2	13.5	1+	plant	90	arboreal	6.4	closed	1+	small	10016822	0.14
Pf	*Pteronotus fulvus*	7	9.21	1	insect	0	aerial	8.3	NA	1	large	3471860	0.20
Pm	*Pteronotus mesoamericanus*	42	19.7	1	insect	0	aerial	6.7	closed	1+	large	539713	0.20
Ra	*Rhogeessa aeneus*	1	4.2	1+	insect	0	aerial	6.1	closed	1	large	144232	0.08
Rn	*Rhynchonycteris naso*	16	3.8	1+	insect	0	aerial	6.5	open	1	medium	11508828	0.45
Sb	*Saccopteryx bilineata*	18	7.8	1	insect	0	aerial	6.1	open	1	medium	12632095	0.45
Sp	*Sturnira parvidens*	47	19.1	1+	plant	100	arboreal	6.5	open	1	small	4859514	0.30
Tc	*Trachops cirrhosus*	8	37.67	1	carnivore	10	ground/aquatic	6.3	closed	1+	small	12642872	0.20
Uc	*Uroderma convexum*	10	16.69	1+	plant	90	arboreal	6.3	NA	1	medium	12796601	0.14

[*]*N*: Number of individuals per bat species screened for hemoplasma infection; AF: annual fecundity; AR: aspect ratio; RF: roost flexibility; ED: evolutionary distinctiveness. Data for *Pf*, *Sp*, *Mn*, *Mp*, and *Uc* were taken from the bat species from which they were originally classified (*Pteronotus davyi*, *Sturnira lilium*, *Molossus rufus, Myotis keaysi*, and *Uroderma bilobatum*).

S3. Infection status and prevalence

Table S3. Hemoplasma infection prevalence (i.e., the proportion of sequence-confirmed, hemoplasma-positive bats) for each species, stratified by site and year in northern Belize.

Species	Site	Year	*N*	+	%
Artibeus intermedius	KK	2018	1	1	1
Artibeus intermedius	LAR	2017	3	2	0.67
Artibeus intermedius	LAR	2018	2	1	0.5
Artibeus jamaicensis	KK	2017	1	1	1
Artibeus jamaicensis	LAR	2017	4	4	1
Artibeus jamaicensis	LAR	2018	6	4	0.67
Artibeus lituratus	KK	2018	1	0	0
Artibeus lituratus	LAR	2017	3	2	0.67
Artibeus lituratus	LAR	2018	4	3	0.75
Bauerus dubiaquercus	LAR	2017	4	0	0
Bauerus dubiaquercus	LAR	2018	2	0	0
Carollia perspicillata	KK	2017	2	1	0.5
Carollia perspicillata	KK	2018	1	0	0
Carollia perspicillata	LAR	2017	3	1	0.33
Carollia perspicillata	LAR	2018	2	1	0.5
Carollia sowelli	KK	2018	1	1	1
Carollia sowelli	LAR	2017	11	2	0.18
Carollia sowelli	LAR	2018	8	4	0.5
Chrotopterus auritus	KK	2017	3	0	0
Chrotopterus auritus	LAR	2018	1	0	0
Dermanura phaeotis	KK	2018	3	0	0
Dermanura phaeotis	LAR	2017	9	4	0.44
Dermanura phaeotis	LAR	2018	7	4	0.57
Dermanura watsoni	KK	2017	1	1	1
Dermanura watsoni	KK	2018	2	1	0.5
Dermanura watsoni	LAR	2017	6	3	0.5
Dermanura watsoni	LAR	2018	5	0	0
Desmodus rotundus	KK	2017	21	20	0.95
Desmodus rotundus	KK	2018	29	14	0.48
Desmodus rotundus	LAR	2017	22	13	0.59
Desmodus rotundus	LAR	2018	68	38	0.56
Eptesicus furinalis	KK	2017	1	1	1
Eptesicus furinalis	LAR	2017	8	6	0.75
Eptesicus furinalis	LAR	2018	10	8	0.8
Eumops nanus	LAR	2017	1	0	0
Gardnerycteris keenani	LAR	2018	2	0	0
Glossophaga soricina	LAR	2017	11	2	0.18
Glossophaga soricina	LAR	2018	7	2	0.29

Lasiurus ega	LAR	2018	3	0	0
Lophostoma evotis	LAR	2017	1	1	1
Lophostoma evotis	LAR	2018	2	2	1
Micronycteris schmidtorum	LAR	2017	1	0	0
Mimon cozumelae	KK	2018	5	0	0
Mimon cozumelae	LAR	2017	1	0	0
Molossus nigricans	LAR	2017	14	13	0.93
Molossus nigricans	LAR	2018	7	3	0.43
Mormoops megalophylla	KK	2018	1	0	0
Mormoops megalophylla	LAR	2018	1	0	0
Myotis elegans	LAR	2018	2	2	1
Myotis pilosatibialis	LAR	2017	1	1	1
Myotis pilosatibialis	LAR	2018	3	2	0.67
Natalus mexicanus	LAR	2018	1	1	1
Noctilio leporhinus	LAR	2018	1	0	0
Platyrrhinus helleri	KK	2018	1	0	0
Platyrrhinus helleri	LAR	2018	1	1	1
Pteronotus fulvus	KK	2017	1	0	0
Pteronotus fulvus	LAR	2017	4	1	0.25
Pteronotus fulvus	LAR	2018	2	1	0.5
Pteronotus mesoamericanus	KK	2017	12	7	0.58
Pteronotus mesoamericanus	KK	2018	6	5	0.83
Pteronotus mesoamericanus	LAR	2017	11	8	0.73
Pteronotus mesoamericanus	LAR	2018	13	9	0.69
Rhogeesa aeneus	LAR	2018	1	0	0
Rhynchonycteris naso	LAR	2017	5	1	0.2
Rhynchonycteris naso	LAR	2018	11	1	0.09
Saccopteryx bilineata	LAR	2017	9	1	0.11
Saccopteryx bilineata	LAR	2018	9	1	0.11
Sturnira parvidens	KK	2017	2	1	0.5
Sturnira parvidens	KK	2018	4	2	0.5
Sturnira parvidens	LAR	2017	23	11	0.48
Sturnira parvidens	LAR	2018	20	13	0.65
Trachops cirrhosus	KK	2017	3	2	0.67
Trachops cirrhosus	KK	2018	1	1	1
Trachops cirrhosus	LAR	2018	4	4	1
Uroderma convexum	LAR	2017	2	1	0.5
Uroderma convexum	LAR	2018	8	3	0.38

Table S4. Competing phylogenetic GLMMs predicting hemoplasma infection status across the Belize bat community (*n*=323 after removing missing values). Models are ranked by ΔLOOIC with their LOOIC SE, Akaike weights (w_i) and Bayesian R^2 estimates.

**Model structure**	**LOOIC**	**SE**	**ΔLOOIC**	w_i	R^2_m	R^2_c
~ sex + ectoparasite + reproduction + site * year + (1|species) + (1|phylogeny)	404.56	16.1	0.00	0.29	0.07	0.23
~ sex + ectoparasite + reproduction + year + (1|species) + (1|phylogeny)	404.72	15.2	0.16	0.27	0.05	0.22
~ sex + ectoparasite + reproduction + site + year + (1|species) + (1|phylogeny)	405.77	15.3	1.21	0.16	0.05	0.22
~ sex * reproduction + ectoparasite + year + (1|species) + (1|phylogeny)	406.57	15.3	2.00	0.11	0.05	0.22
~ sex * reproduction + ectoparasite + site * year + (1|species) + (1|phylogeny)	406.72	16.2	2.16	0.10	0.07	0.24
~ sex * reproduction + ectoparasite + year + (1|species) + (1|phylogeny)	407.48	15.6	2.92	0.07	0.06	0.22

S4. Hemoplasma genotypes

We here provide details of how each identified genotype (Table 2) is phylogenetically related to one another and other sequences identified in GenBank (updated March 2020, see Fig. S3).

The MR1 genotype, identified from *Molossus nigricans* (formerly classified as *M. rufus* [1]), showed 92.5% and 94.3% similarity to *Candidatus* Mycoplasma turicensis and *Mycoplasma coccoides*, as well as 98.3% similarity to hemoplasmas previously detected in *M. molossus* in Brazil (KY356748) [2]. The PPM genotype, which had 98.6% similarity to VBG1 [3], was identified from *Pteronotus mesoamericanus* and in one sample from *P. fulvus* (formerly classified as *P. davyi* [4,5]; MK353825). The 16S rRNA sequence from another *P. fulvus* (MH245133) was identical to VBG1. The NM genotype, identified in one *Natalus mexicanus*, showed 99.3% and 98.3% similarity to PPM and VBG1, respectively; however, the species-specific pattern of mutations allowed us to discriminate NM from these two genotypes. The RHN genotype, identified in a single *Rhynchonycteris naso*, demonstrated a unique sequence with 93.3-94.4% similarity to *Candidatus* Mycoplasma haemohominis [6], *Candidatus* Mycoplasma turicensis, and *M. haemomuris*; 94.8% similarity to *Candidatus* Mycoplasma haemomacaque; and 95.6-96.1% similarity to the PPM and SP genotypes (see below).

Two distinct hemoplasma genotypes (CS1 and CS2) were identified in *Carollia* spp. CS1, only identified in *C. sowelli*, was 98.0% similar to VBG3, while CS2 (identified in both *C. sowelli* and *C. perspicillata*) was 97.7% and 97.4% similar to VBG3 and CS1, respectively. The unique GLS genotype identified in a single *Glossophaga soricina* demonstrated 98.1% similarity to VBG3 and 96.8% and 97.4% similarity to the CS1 and CS2 genotypes, respectively.

The EF1 genotype comprised hemoplasma sequences that were mainly identified in *Eptesicus furinalis* alongside single positive samples from *Saccopteryx bilineata* (MK353889)

and *Glossophaga soricina* (MH245128); this genotype was 96.9% similar to *Candidatus* M. haemohominis as well as 96.7-98.2% similar to hemoplasma genotypes previously detected in *Miniopterus schreibersii* and *Myotis capaccinii* from Spain [7] and to a hemoplasma genotype previously detected in bat ticks (*Ixodes simplex*) from Hungary [8]. The MYK genotype, identified in one *Myotis pilosatibialis* (previously classified as *M. keaysi* [9]), was 97.3% similar to the EF1 genotype , 96.2% similar to *Candidatus* M. haemohominis, and 96.5-98.0% similar to the above-mentioned genotypes previously detected in bats from Spain [7] and bat ticks from Hungary [8]. Both the EF2 genotype (identified in *E. furinalis*) and the MR2 genotype (identified in *Molossus nigricans*) were 98.9% similar to one another; however, species-specific mutation patterns again facilitated discriminating these genotypes. Both genotypes were 97.7% similar to the MYE genotype from *Myotis elegans* and *M. pilosatibialis*, and all three of these genotypes (MYE, EF2, and MR2) showed 97.6-98.3% similarity to the hemoplasma genotype previously identified in little brown bats (*M. lucifugus*) from the eastern and northeastern United States [10] and to hemoplasmas detected in *M. chiloensis* from Chile (MK295627, MK295629, and MK295630) [11]. The EF2 and MR2 genotypes were also 93.5-94.5% similar to *Candidatus* M. haemomacaque, *M. haemomuris, Candidatus* M. turicensis, and *Candidatus* M. haemohominis.

The APH1 genotype was identified in *Dermanura phaeotis, D. watsoni*, and in one *A. lituratus*; this genotype showed 99.0% similarity to the PLU genotype detected in one *Platyrrhinus helleri* and in one *Uroderma convexum* (formerly classified as *U. bilobatum* [12,13]). These genotypes were further discriminated based on species-specific pattern of their mutations. The APH1 and PLU genotypes were 96.3-96.6% similar to the APH2 genotype from *Artibeus jamaicensis, A. lituratus*, and *A. intermedius*. The APH2 genotype showed 98.3%

similarity to VBG2 [3] and the UB genotype. The APH3 genotype was found in *A. intermedius* and was 96.2-96.7% similar to the CS1 and CS2 genotypes.

Two genotypes (TC1 and TC2) identified in *Trachops cirrhosus* were 96.7% similar to each other, and both were 96.6% similar to the LE genotype identified in *Lophostoma evotis*. The TC1, TC2, and LE genotypes were 97.3%, 96.3%, and 96.6% similar to the PPM and NM genotypes. The SP genotype, which comprised slightly divergent (99.4% intra-genotype sequence similarity) but closely related hemoplasma species, was identified in *Sturnira parvidens* (previously classified as *S. lilium* [14,15]) and in one *A. lituratus*. The SP genotype was most closely related to the UB genotype (99.1% similar) and was 98.4% similar to the APH2 genotype and VBG2. However, due to some diversity of SP sequences and their similarity to UB, APH2, and VBG2, the SP genotype did not form a distinct branch with significant bootstrap support on the phylogeny (Fig. S3). Thus, sequences within the SP genotype may comprise at least three closely related hemoplasma species or geographic clones (or isolates) of the same species (e.g., see the hypothetical groups A, B, and C on Fig. S3). However, definitive conclusions on the hemoplasma species repertoire within the SP genotype cannot be made given only analysis of the partial 16S rRNA gene; the nucleotide and phylogenetic analysis of additional housekeeping genes (e.g., *rpoB, gyrB*) [16,17] or the employment of next-generation sequencing methods [18–20] to screen for the full spectrum of blood-borne bacteria in bat samples will be required for future studies of these hemoplasma genotypes. All genotypes demonstrated minor levels of intra-genotype sequence variability (99.4–100%; see Table 2).

Lastly, three non-hemotropic *Mycoplasma* spp. genotypes were identified from five different bat species in Belize. Three *M. moatsii*–like sequences (numbered as 1–3) from *Pteronotus mesoamericanus, Myotis pilosatibialis*, and *Rhynchonycteris naso* showed 95.2%,

98.1%, and 96.9% similarity, respectively, to the 16S rRNA gene of *M. moatsii* type strain MK405 (NR_025186), a non-hemotropic *Mycoplasma sp.* isolated from grivet monkeys (*Cercopithecus aethiops*) [21]. We previously reported the *M. moatsii*–like genotype in *Desmodus rotundus* (KY932724) from Belize sampled in 2014 and 2015 [3]. However, these three novel *M. moatsii*–like sequences were not identical to each other or to the *M. moatsii*–like genotype previously found in *D. rotundus* (numbered as 4)*;* thus, the *M. moatsii*–like genotype 1 showed 97.0%, 96.1%, and 96.9% similarity to the *M. moatsii*–like genotypes 2-4, the *M. moatsii*–like genotype 2 showed 97.0%, 97.9%, and 98.2% similarity to the *M. moatsii*–like genotypes 1, 3-4, the *M. moatsii*–like genotype 3 showed 96.1%, 97.9%, and 96.3% similarity to the *M. moatsii*–like genotypes 1, 2, 4. This suggests these sequences are derived from closely related but different non-hemotropic *Mycoplasma* spp. in bats. The *M. lagogenitalium*-like genotype from *G. soricina* showed 96.5% and 97.6% similarity to the 16S rRNA genes of *M. lagogenitalium* type strain 12MS (NR_025185), a non-hemotropic *Mycoplasma sp.* described in Afghan pikas (*Ochotona rufescens*) [22] and *Mycoplasma* sp. EDS-4 isolated from house musk shrew (*Suncus murinus*) [23]. The *M. muris*–like genotype identified from *Saccopteryx bilineata* demonstrated 98.1% similarity to the 16S rRNA genes of *M. muris* type strain RIII-4 (NR_044664), a non-hemotropic *Mycoplasma* sp. isolated from mice [24].

References

1. Loureiro LO, Engstrom MD, Lim BK. 2020 Single nucleotide polymorphisms (SNPs) provide unprecedented resolution of species boundaries, phylogenetic relationships, and genetic diversity in the mastiff bats (Molossus). *Mol. Phylogenet. Evol.* **143**, 106690.

2. Ikeda P *et al.* 2017 Evidence and molecular characterization of Bartonella spp. and hemoplasmas in neotropical bats in Brazil. *Epidemiol. Amp Infect.* , 1–15. (doi:10.1017/S0950268817000966)

3. Volokhov DV, Becker DJ, Bergner LM, Camus MS, Orton RJ, Chizhikov VE, Altizer SM, Streicker DG. 2017 Novel hemotropic mycoplasmas are widespread and genetically diverse in vampire bats. *Epidemiol. Infect.*

4. Pavan AC, Marroig G. 2016 Integrating multiple evidences in taxonomy: species diversity and phylogeny of mustached bats (Mormoopidae: Pteronotus). *Mol. Phylogenet. Evol.* **103**, 184–198.

5. Pavan AC, Marroig G. 2017 Timing and patterns of diversification in the Neotropical bat genus Pteronotus (Mormoopidae). *Mol. Phylogenet. Evol.* **108**, 61–69.

6. Steer JA, Tasker S, Barker EN, Jensen J, Mitchell J, Stocki T, Chalker VJ, Hamon M. 2011 A Novel Hemotropic Mycoplasma (Hemoplasma) in a Patient With Hemolytic Anemia and Pyrexia. *Clin. Infect. Dis. Off. Publ. Infect. Dis. Soc. Am.* **53**, e147–e151. (doi:10.1093/cid/cir666)

7. Millán J, López-Roig M, Delicado V, Serra-Cobo J, Esperón F. 2015 Widespread infection with hemotropic mycoplasmas in bats in Spain, including a hemoplasma closely related to "Candidatus Mycoplasma hemohominis". *Comp. Immunol. Microbiol. Infect. Dis.* **39**, 9–12. (doi:10.1016/j.cimid.2015.01.002)

8. Hornok S *et al.* 2019 Molecular detection of vector-borne bacteria in bat ticks (Acari: Ixodidae, Argasidae) from eight countries of the Old and New Worlds. *Parasit. Vectors* **12**, 50. (doi:10.1186/s13071-019-3303-4)

9. Mantilla-Meluk H, Munoz-Garay J. 2014 Biogeography and taxonomic status of Myotis keaysi pilosatibialis LaVal 1973 (Chiroptera: Vespertilionidae). *Zootaxa* **3793**, 60–70.

10. Mascarelli PE, Keel MK, Yabsley M, Last LA, Breitschwerdt EB, Maggi RG. 2014 Hemotropic mycoplasmas in little brown bats (Myotis lucifugus). *Parasit Vectors* **7**, 117.

11. Millán J, Cevidanes A, Sacristán I, Alvarado M, Sepúlveda G, Ramos-Mella CA, Lisón F. 2019 Detection and Characterization of Hemotropic Mycoplasmas in Bats in Chile. *J. Wildl. Dis.* (doi:10.7589/2018-12-290)

12. Mantilla-Meluk H. 2014 *Defining species and species boundaries in Uroderma (Chiroptera: Phyllostomidae) with a description of a new species*. Museum of Texas Tech University.

13. Cuadrado-Ríos S, Mantilla-Meluk H. 2016 Timing the evolutionary history of tent-making bats, genus Uroderma (Phyllostomidae): A biogeographic context. *Mamm. Biol.* **81**, 579–586. (doi:10.1016/j.mambio.2016.07.045)

14. Hernández-Canchola G, León-Paniagua L. 2017 Genetic and ecological processes promoting early diversification in the lowland Mesoamerican bat Sturnira parvidens (Chiroptera: Phyllostomidae). *Mol. Phylogenet. Evol.* **114**, 334–345.

15. Velazco PM, Patterson BD. 2013 Diversification of the yellow-shouldered bats, genus Sturnira (Chiroptera, Phyllostomidae), in the New World tropics. *Mol. Phylogenet. Evol.* **68**, 683–698.

16. Volokhov DV, Simonyan V, Davidson MK, Chizhikov VE. 2012 RNA polymerase beta subunit (rpoB) gene and the 16S–23S rRNA intergenic transcribed spacer region (ITS) as complementary molecular markers in addition to the 16S rRNA gene for phylogenetic analysis and identification of the species of the family Mycoplasmataceae. *Mol. Phylogenet. Evol.* **62**, 515–528.

17. Volokhov DV, Hwang J, Chizhikov VE, Danaceau H, Gottdenker NL. 2017 Prevalence, Genotype Richness, and Coinfection Patterns of Hemotropic Mycoplasmas in Raccoons (Procyon lotor) on Environmentally Protected and Urbanized Barrier Islands. *Appl. Environ. Microbiol.* **83**. (doi:10.1128/AEM.00211-17)

18. Horiba K *et al.* 2018 Comprehensive detection of pathogens in immunocompromised children with bloodstream infections by next-generation sequencing. *Sci. Rep.* **8**, 3784. (doi:10.1038/s41598-018-22133-y)

19. Van Borm S *et al.* 2015 Next-generation sequencing in veterinary medicine: how can the massive amount of information arising from high-throughput technologies improve diagnosis, control, and management of infectious diseases? *Methods Mol. Biol. Clifton NJ* **1247**, 415–436. (doi:10.1007/978-1-4939-2004-4_30)

20. Granberg F, Bálint Á, Belák S. 2016 Novel technologies applied to the nucleotide sequencing and comparative sequence analysis of the genomes of infectious agents in veterinary medicine. *Rev. Sci. Tech. Int. Off. Epizoot.* **35**, 25–42. (doi:10.20506/rst.35.1.2415)

21. Madden DL, Moats KE, London WT, Matthew EB, Sever JL. 1974 Mycoplasma moatsii, a new species isolated from recently imported Grivit monkeys (Cercopithecus aethiops). *Int. J. Syst. Evol. Microbiol.* **24**, 459–464.

22. Kobayashi H, Runge M, Schmidt R, Kubo M, Yamamoto K, Kirchhoff H. 1997 Mycoplasma lagogenitalium sp. nov., from the preputial smegma of Afghan pikas (Ochotona rufescens rufescens). *Int. J. Syst. Bacteriol.* **47**, 1208–1211. (doi:10.1099/00207713-47-4-1208)

23. Goto K, Yasuda M, Hayashimoto N, Ebukuro S. 2010 Morphological and sequence analysis of Mycoplasma sp. isolated from the oral cavity of a house musk shrew (Suncus murinus). *J. Vet. Med. Sci.* **72**, 109–111.

24. McGarrity GJ, Rose DL, Kwiatkowski V, Dion AS, Phillips DM, Tully JG. 1983 Mycoplasma muris, a new species from laboratory mice. *Int. J. Syst. Evol. Microbiol.* **33**, 350–355.

Figure S3. Phylogenetic relationships based on sequence data for the partial 16S rRNA gene (approx. 864-878 bp) among the hemoplasma genotypes detected in Belize bat species with other hemotropic and non-hemotropic *Mycoplasma* spp. Accession numbers are shown alongside bat species names. The tree was constructed with the minimum evolution method in MEGA X. Bootstrap values were evaluated from 1000 replications; those < 50% are not included on the tree. *Streptococcus equi* is used as an outgroup. The bar indicates 0.05 substitutions per site.

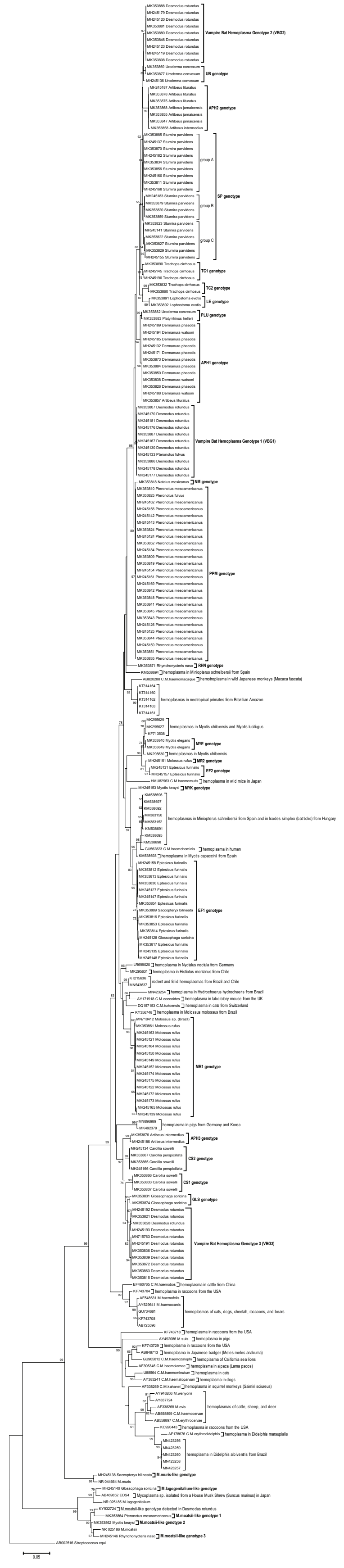
Vampire Bat Hemoplasma Genotype 2 (VBG2)
UB genotype
APH2 genotype
group A
group B
group C
SP genotype
TC1 genotype
TC2 genotype
LE genotype
PLU genotype
APH1 genotype
Vampire Bat Hemoplasma Genotype 1 (VBG1)
NM genotype
PPM genotype
RHN genotype
MYE genotype
MR2 genotype
EF2 genotype
MYK genotype
EF1 genotype
MR1 genotype
APH3 genotype
CS2 genotype
CS1 genotype
GLS genotype
Vampire Bat Hemoplasma Genotype 3 (VBG3)
M.muris-like genotype
M.lagogenitalium-like genotype
M.moatsii-like genotype 1
M.moatsii-like genotype 2
M.moatsii-like genotype 3
0.05

Figure S4. Relative abundance of the 29 hemoplasma genotypes detected in the Belize Neotropical bat community in northern Belize according to site, year, and bat species.

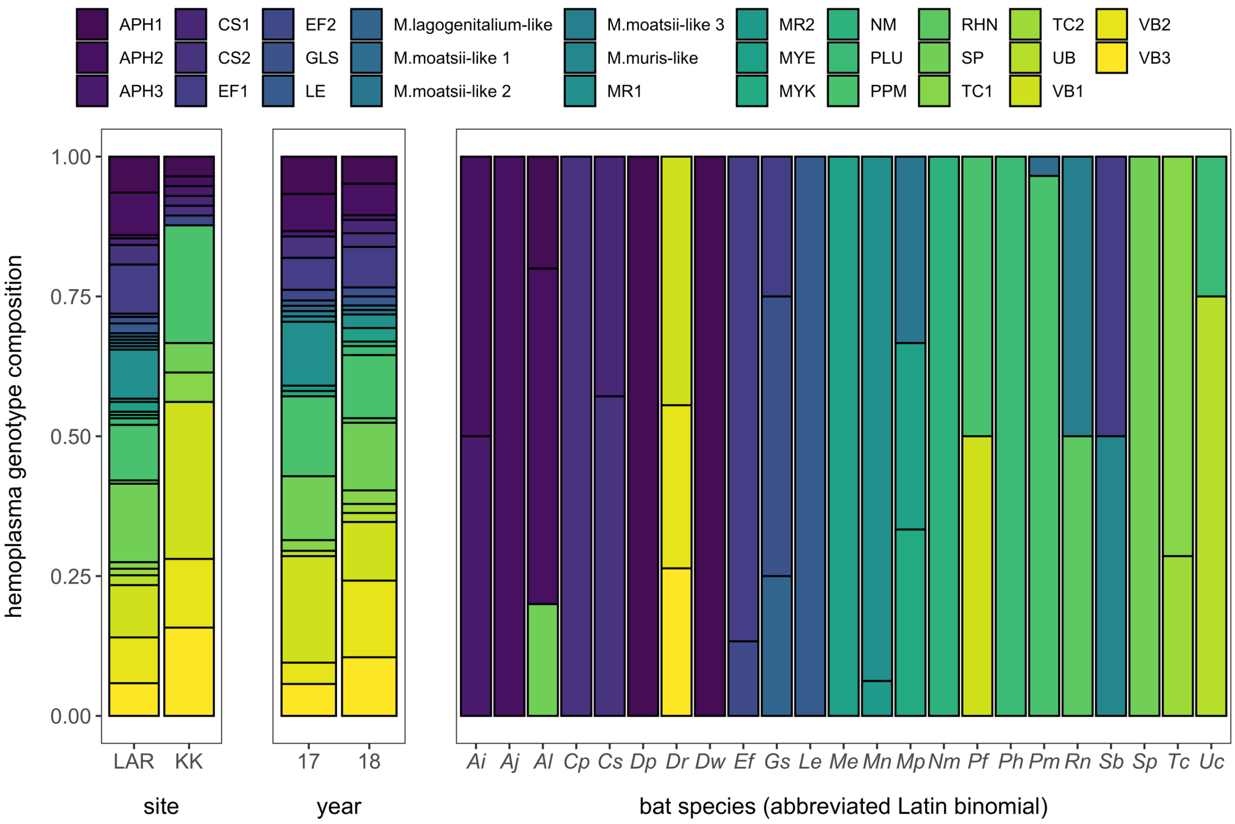

Figure S5. Contribution of unique bat–genotype links (squared residuals) to cophylogenetic signal (m^2_{XY}) from PACo. Links are considered supportive of coevolution if the upper 95% confidence interval falls below the mean squared residuals (shown in the dashed line).

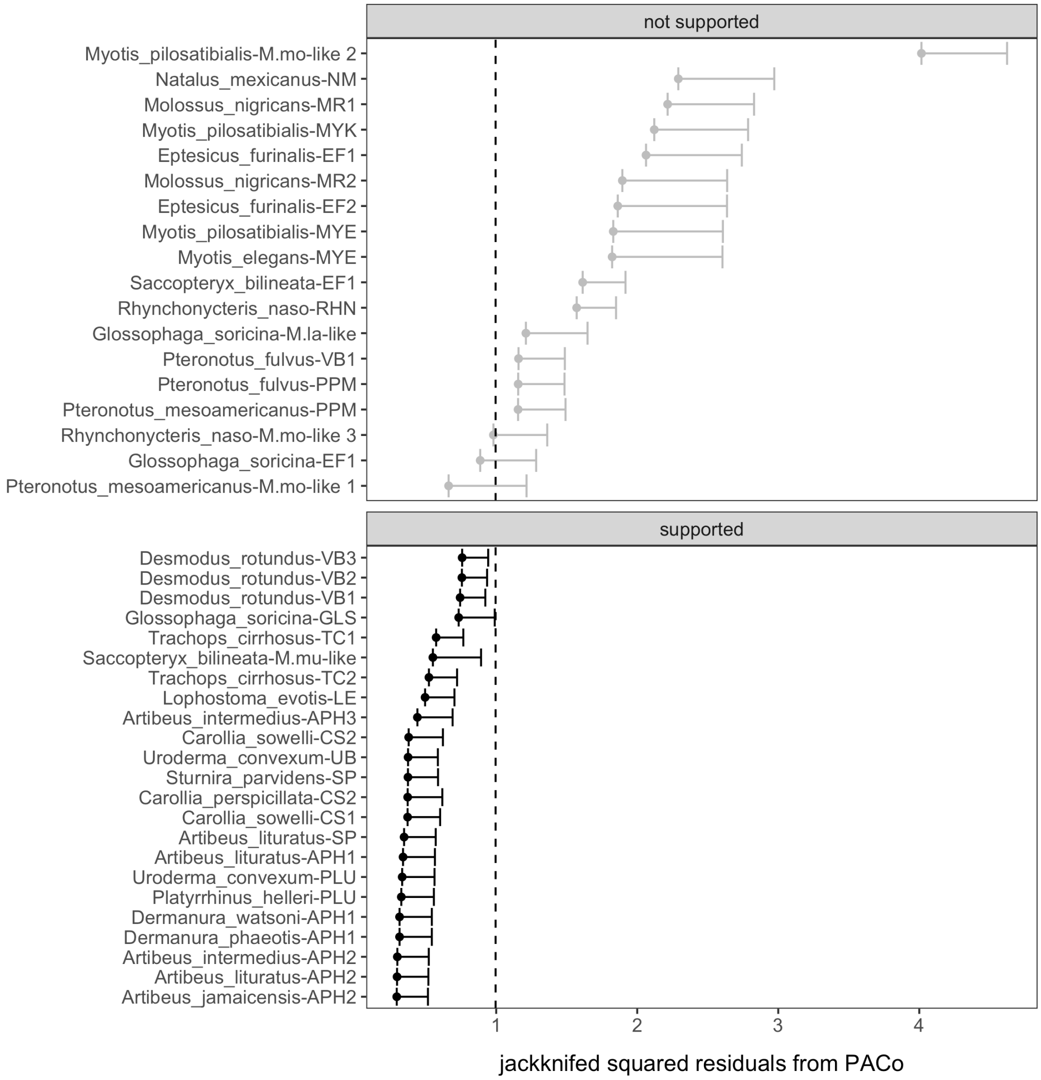

Figure S6. Metrics of network centrality (degree, eigenvector centrality) for sharing of hemoplasma genotypes between bat species in Belize.

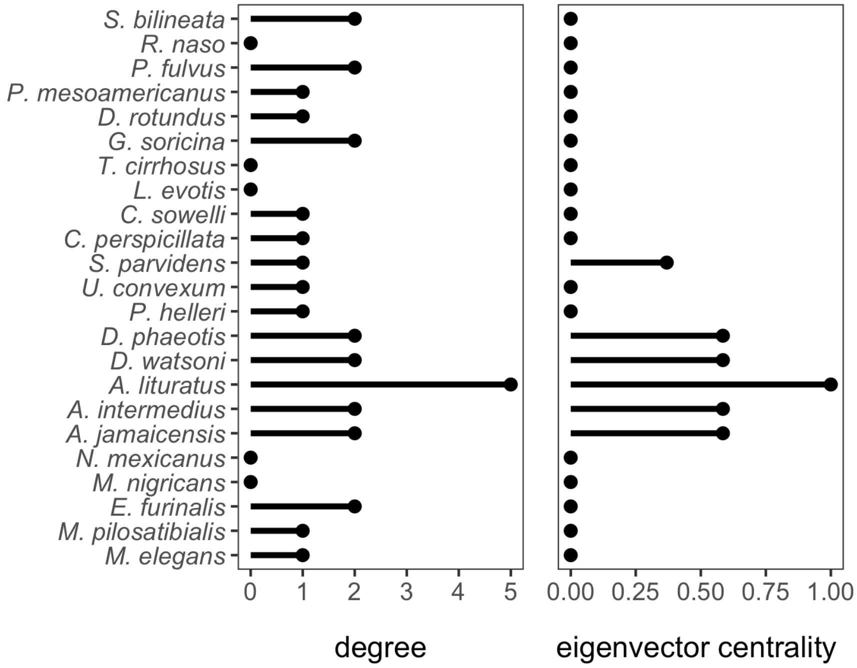

Figure S7. Networks of hemoplasma genotype sharing across the Belize bat community, stratified by site and year. Nodes represent bat species (abbreviated by their Latin binomial), and edges represent whether two nodes share any hemoplasma genotypes. Nodes with no edges represent bat species that do not share any hemoplasma genotypes with other bat species.

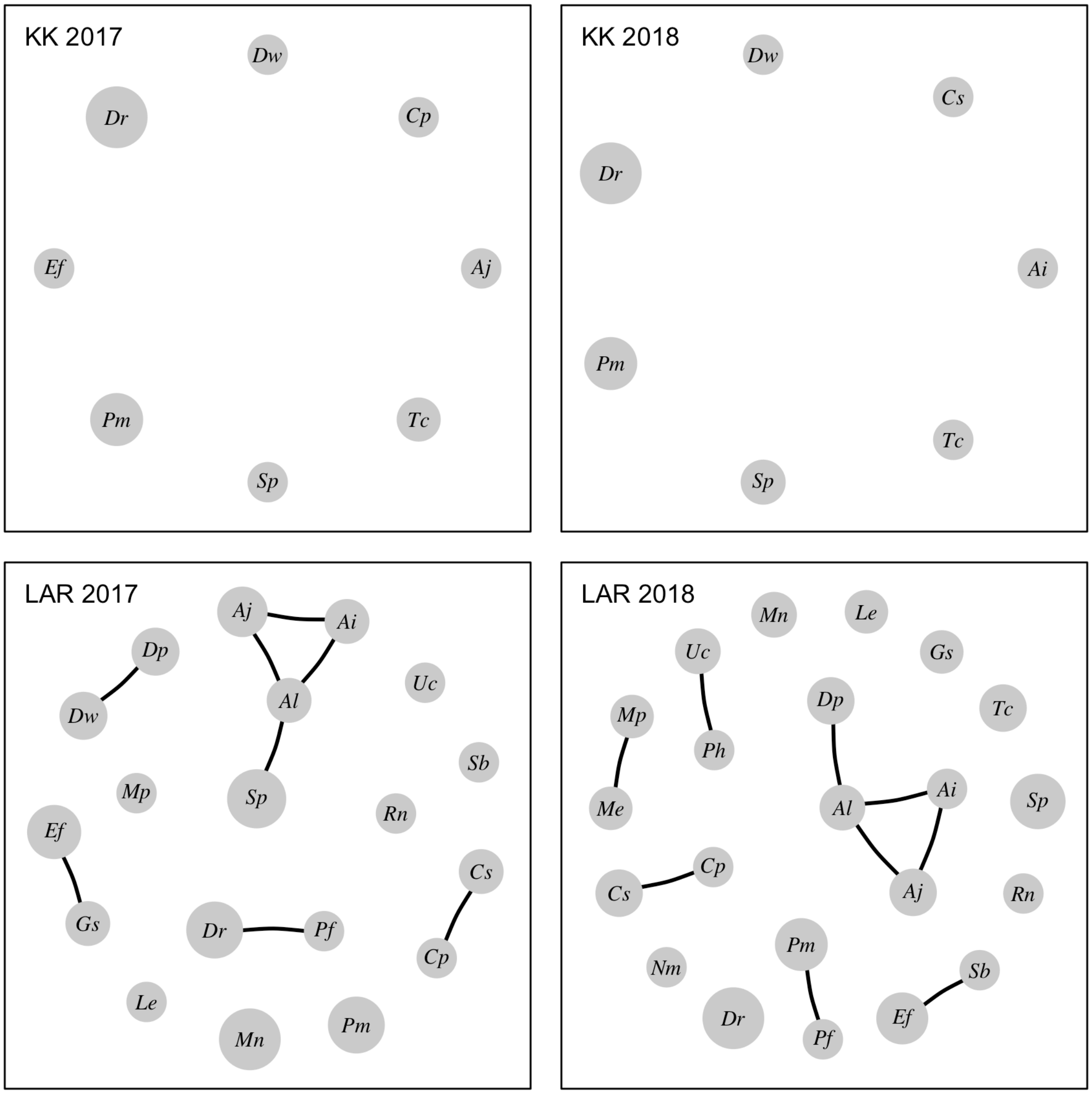

Table S5. Results of GLMs applied to each hemoplasma genotype sharing network centrality measure as a function of site, year, and their interaction term.

**Variable**	χ^2	***p***
Degree		
Site	21.19	<0.001
Year	0.007	0.93
Site * year	0	1
Eigenvector centrality		
Site	112.33	<0.001
Year	0.046	0.83
Site * year	0.017	0.90

Table S6. Competing PGLS models predicting degree (from the hemoplasma genotype network) across the Belize bat community. Models are ranked by ΔAICc with the number of coefficients (*k*), Akaike weights (w_i), and a likelihood ratio test pseudo-R^2.

**Model structure**	***k***	**ΔAICc**	w_i	R^2
Square-root degree ~ % plants	2	0	0.25	0.21
Square-root degree ~ log aspect ratio	2	0.82	0.16	0.18
Square-root degree ~ dietary guild	3	1.66	0.11	0.25
Square-root degree ~ log annual fecundity	2	2.02	0.09	0.14
Square-root degree ~ log evolutionary distinctiveness	2	2.12	0.08	0.13
Square-root degree ~ 1	1	2.77	0.06	0
Square-root degree ~ roost flexibility	2	3.35	0.05	0.09
Square-root degree ~ foraging strata	3	3.51	0.04	0.19
Square-root degree ~ colony size	2	3.59	0.04	0.08
Square-root degree ~ log mass	2	3.76	0.04	0.07
Square-root degree ~ square-root geographic range	2	4.09	0.03	0.06
Square-root degree ~ roost type	2	4.1	0.03	0.06
Square-root degree ~ sample size	2	5.42	0.02	0

Table S7. Competing PGLS models predicting eigenvector centrality (from the hemoplasma genotype network) across the Belize bat community. Models are ranked by ΔAICc with the number of coefficients (*k*), Akaike weights (w_i), and a likelihood ratio test pseudo-R^2.

**Model structure**	***k***	**ΔAICc**	w_i	R^2
Logit eigenvector centrality ~ colony size	2	0	0.24	0.13
Logit eigenvector centrality ~ 1	1	0.61	0.18	0
Logit eigenvector centrality ~ % plants	2	0.87	0.16	0.1
Logit eigenvector centrality ~ log evolutionary distinctiveness	2	2.22	0.08	0.04
Logit eigenvector centrality ~ roost type	2	3.04	0.05	0.01
Logit eigenvector centrality ~ log mass	2	3.21	0.05	0
Logit eigenvector centrality ~ sample size	2	3.27	0.05	0
Logit eigenvector centrality ~ roost flexibility	2	3.28	0.05	0
Logit eigenvector centrality ~ log annual fecundity	2	3.65	0.04	0
Logit eigenvector centrality ~ square-root geographic range	2	3.8	0.04	0
Logit eigenvector centrality ~ log aspect ratio	2	3.82	0.04	0
Logit eigenvector centrality ~ dietary guild	3	4.34	0.03	0.08
Logit eigenvector centrality ~ foraging strata	3	4.87	0.02	0.06

Figure S8. Associations between hemoplasma genotype sharing network centrality metrics and hemoplasma infection prevalence (logit-transformed as the response variable). PGLS model fit and 95% confidence intervals are shown overlaid with bat species data scaled by sample size.

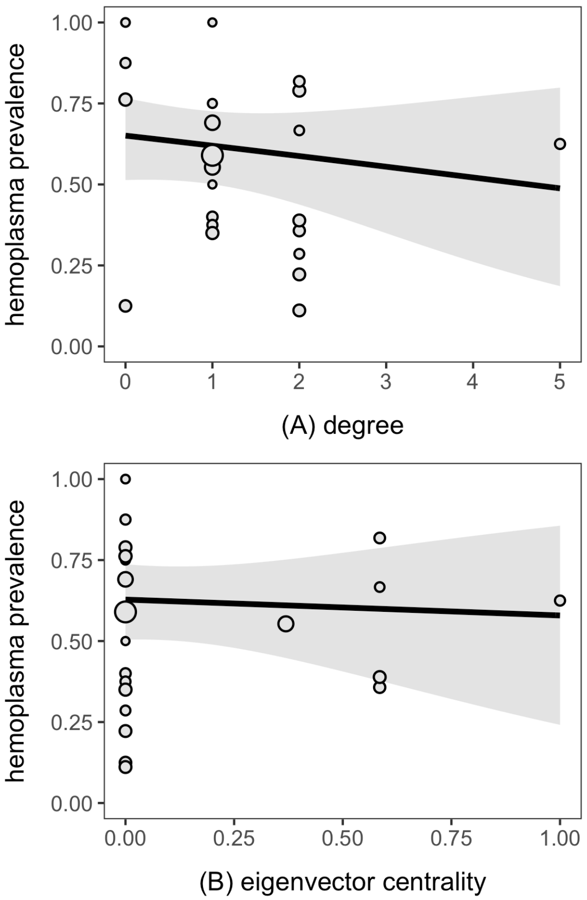

S5. Ectoparasitism

Figure S9. Predictors of individual ectoparasite presence. Odds ratios and 95% HDIs from the phylogenetic GLMMs, including the univariate sex effect and that adjusted for other covariates. Reference levels include males, bats sampled at LAR, reproductive bats, and bats from 2017.

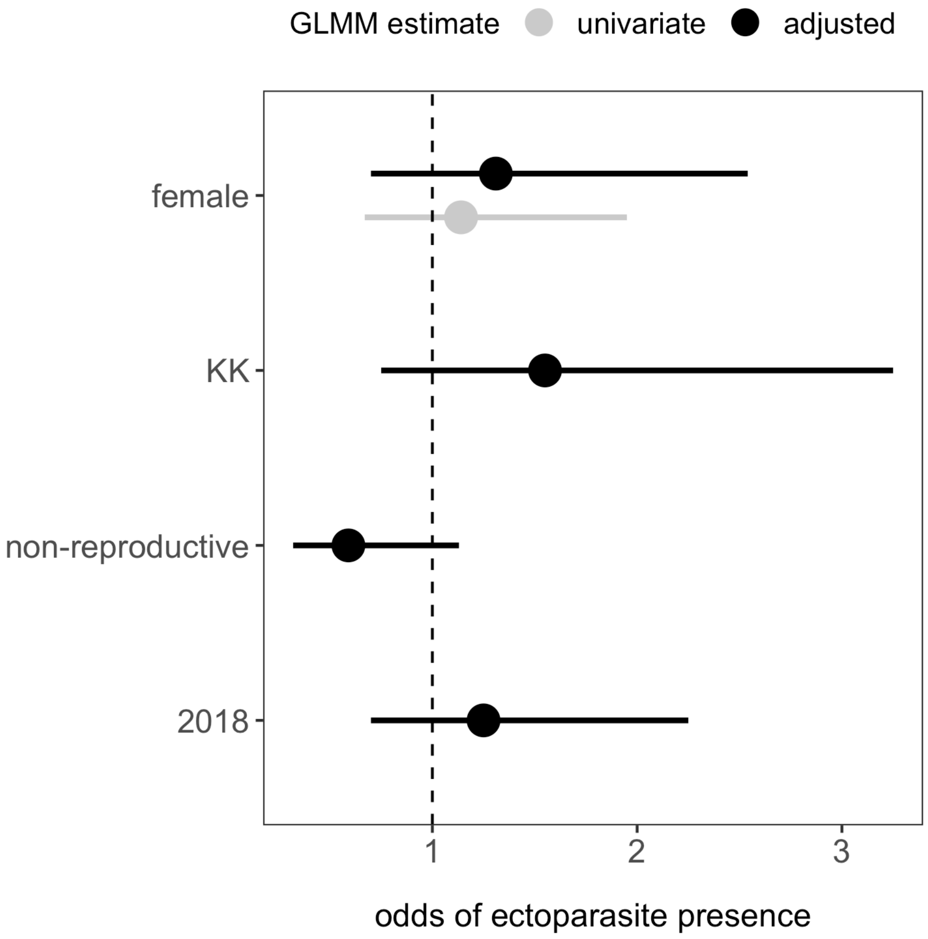

Supplement: Supplementary file 1 — Supplementary Material [file MEC-29-1534-s001.pdf]
